# Spontaneous formation of a self-healing carbon nanoskin at the liquid–liquid interface

**DOI:** 10.1038/s41467-022-31277-5

**Published:** 2022-08-23

**Authors:** Enzo Bomal, Paul Grandgeorge, Reuben J. Yeo, Nicolas Candau, Pedro M. Reis, Holger Frauenrath

**Affiliations:** 1 Ecole Polytechnique Fédérale de Lausanne (EPFL), Institute of Materials, Laboratory of Macromolecular and Organic Materials, EPFL–STI–IMX–LMOM, MXG 037, Station 12, 1015 Lausanne, Switzerland; 2 Ecole Polytechnique Fédérale de Lausanne (EPFL), Institute of Mechanical Engineering, Flexible Structures Laboratory, EPFL–STI–IGM–FLEXLAB, ME D0 1226, Station 9, 1015 Lausanne, Switzerland; 3 Present Address: Materials Science and Engineering, University of Washington, Seattle, WA USA; 4 Present Address: Institute of Materials Research and Engineering, 2 Fusionopolis Way, Innovis 08-03, Singapore, 138634 Singapore; 5 Present Address: Centre Català del Plàstic, Universitat Politècnica de Catalunya Barcelona Tech, Av. D’Eduard Maristany, 16, 08019 Barcelona, Spain

**Keywords:** Molecular self-assembly, Self-assembly, Synthesis and processing

## Abstract

Biological membranes exhibit the ability to self-repair and dynamically change their shape while remaining impermeable. Yet, these defining features are difficult to reconcile with mechanical robustness. Here, we report on the spontaneous formation of a carbon nanoskin at the oil–water interface that uniquely combines self-healing attributes with high stiffness. Upon the diffusion-controlled self-assembly of a reactive molecular surfactant at the interface, a solid elastic membrane forms within seconds and evolves into a continuous carbon monolayer with a thickness of a few nanometers. This nanoskin has a stiffness typical for a 2D carbon material with an elastic modulus in bending of more than 40–100 GPa; while brittle, it shows the ability to self-heal upon rupture, can be reversibly reshaped, and sustains complex shapes. We anticipate such an unusual 2D carbon nanomaterial to inspire novel approaches towards the formation of synthetic cells with rigid shells, additive manufacturing of composites, and compartmentalization in industrial catalysis.

## Abstract (web summary)

Biological membranes exhibit the ability to self-repair and dynamically change their shape while remaining impermeable but these defining features are difficult to reconcile with mechanical robustness. Here, the authors report on the spontaneous formation of a carbon nanoskin at the oil-water interface that uniquely combines self-healing attributes with high stiffness.

## Introduction

A fundamental aspect of biological systems is their ability to compartmentalize liquids with mechanically stable membranes^1^. While biological membranes can exhibit some degree of solid-like behavior and withstand mechanical loading^2^, they also show the ability to dynamically reshape upon external stimuli^3^ and self-repair^4^, that is, features typically associated with fluid behavior. Synthetic surfactants and colloidal assemblies can mimic some of these features to create tissue-like networks^5^, or active droplets that are responsive to stimuli, including light^6^, temperature, electric, or magnetic fields^7^. A key aspect of these functional interfaces is their mechanical resilience, which prevents coalescence^8^ and allows for the stabilization of complex shapes that would be thermodynamically out of equilibrium for purely liquid systems. An improved understanding of mechanically stable interfacial membranes has contributed to recent advances in chemical technology, such as biphasic-continuous reactive separation with large interfacial area^9^ and additive manufacturing using microdroplets^10^.

Mechanically more robust interfaces can be achieved by the co-assembly of colloids and polymers at the liquid–liquid interface, relying on the effect of interfacial jamming^11^. Russell et al. have used this approach to create structured liquids of various shapes and sizes within a second liquid phase^12^. Carbon nanomaterials, with their combination of high stiffness and conductivity^13,14^, have been prepared using liquid-fluid interfaces as templates^15,16^ and have emerged as promising building blocks for the creation of mechanically robust, complex interfaces^17^. Graphene oxide, for instance, has been used as a model to study the assembly of 2D nanomaterials at the liquid–liquid interface where its barrier properties have significantly reduced the evaporation of the oil phase^18^.

**Fig. 1:**
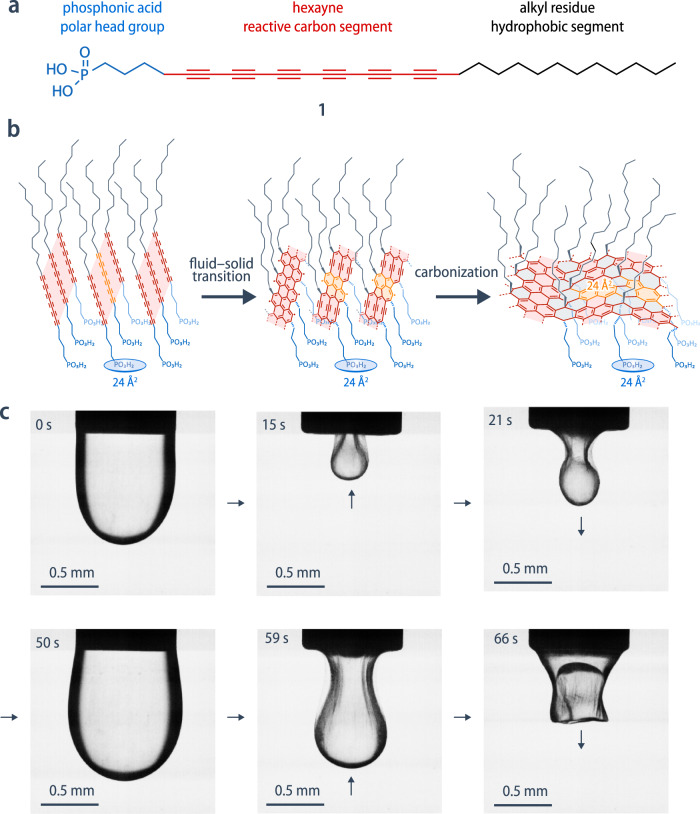
The fluid-solid transition at the interface. **a** Molecular structure of the carbon-rich surfactant **1** (octacosa-5,7,9,11,13,15-hexayne phosphonic acid) used for the spontaneous formation of the carbon nanoskin. **b** Illustration of the proposed interfacial carbonization of the surfactant **1** by 2D polymerization of the twelve hexayne carbon atoms via a cross-linked poly(ene-yne) to the final amorphous carbon monolayer constituted of different C_12_ “carbon tiles” whose average size is similar to the mean molecular area of the densely packed phosphonate groups. **c** Macroscopic wrinkles appear upon deflation of a droplet of a chloroform solution of surfactant **1** in water, revealing the solid nature of the formed membrane. The macroscopic droplet can be held in place by the nanoskin, re-inflated back to its original shape, and deflated once again.

Here, we demonstrate how the liquid–liquid interface can be used as a template for the spontaneous formation of an ultrathin but stiff carbon nanoskin that represents the first example of a carbon nanomaterial with the biomimetic ability to self-repair and be reversibly reshaped. Starting from a dilute solution of the reactive hexayne phosphonic acid surfactant **1** as the single component, the spontaneous solidification of the interface is triggered once a critical interfacial concentration is surpassed and proceeds with a low activation barrier because the mean molecular area of **1** matches the microstructure of the fully carbonized end product (Fig. 1a, b).

## Results and discussion

### Interfacial self-assembly and carbonization

We have monitored the time evolution of the interfacial adsorption of the reactive carbon-rich surfactant **1**^19^ at the chloroform-water interface by measuring the equilibrium interfacial tension, *γ*, at different concentrations, *c*, using the pendant drop method^20^. At low concentrations (*c* ≤ 0.01 mmol/L), the equilibrium interfacial tension remains constant at *γ* ≈ 27 mN/m, close to the value of the pure chloroform/water interface ($\bar{\gamma }=$ 30 mN/m). A sharp decrease in γ occurs at *c* ≈ 0.02 mmol/L, and for *c* ≥ 0.05 mmol/L, the equilibrium interfacial tension levels off towards *γ* ≈ 5 mN/m (Supplementary Fig. 1), indicating the saturation of the interface with surfactant. Interestingly, we consistently observe wrinkling upon interfacial area reduction by deflating a droplet containing surfactant **1** (Fig. 1c and Supplementary Movie 1), contrary to control experiments with a regular surfactant and another hexayne surfactant where no wrinkling was observed (Supplementary Fig. 2). This wrinkling suggests the presence of an elastic solid film (skin) at the liquid–liquid interface^21,22^ that is spontaneously formed in a remarkably short time scale of a few seconds of exposing the carbon-rich surfactant **1** to the chloroform-water interface. The resulting skin is sufficiently robust to be rapidly cycled in and out of the syringe multiple times, which occasionally leads to the transient formation of complex shapes such as tubes (Supplementary Fig. 3 and Supplementary Movie 2).

**Fig. 2:**
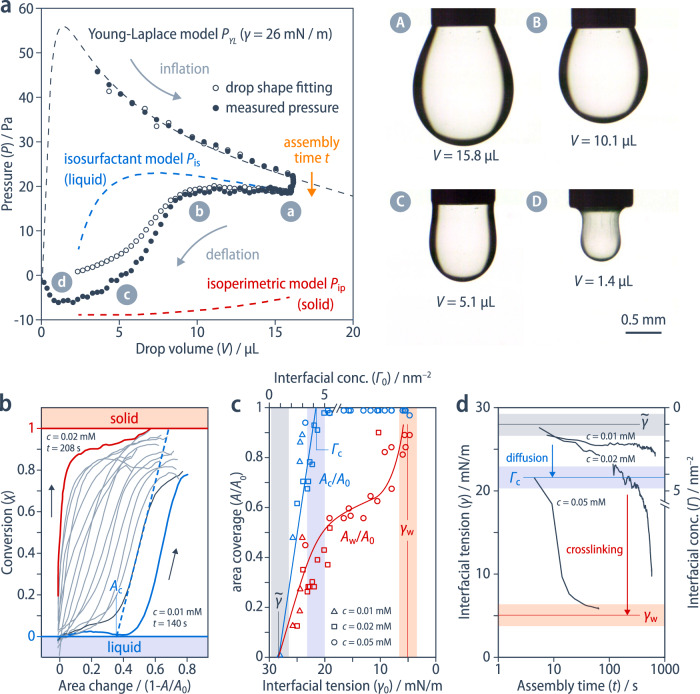
Pressure-volume curve analysis and kinetics of the film formation. **a** Example of a pressure–volume curve of a droplet of a solution of **1** (*c* = 0.02 mmol/L, *t* = 23 s) in water. The experimentally measured pressure (filled symbols) is compared to the pressure inferred from the fitting of the drop shape using the Young–Laplace equation (empty symbols); dashed lines represent the pressure predictions using different models. Images A–D show snapshots of the pendant drop at different stages of deflation. **b** Representative plots of the conversion, *χ*, of the interface from a liquid to a solid state at various assembly times, *t*, and concentrations, *c*, as a function of the relative change in the drop's surface area 1–*A*/*A*_0_; the critical interfacial area, *A*_c_, is defined as the intersection of the tangent at the point of the highest slope with *χ* = 0, representing the onset at which the interface starts to no longer exhibit a liquid behavior. **c** Plot of the relative critical interfacial area *A*_c_/*A*_0_ (blue) and the relative interfacial area at the onset of wrinkling *A*_w_/*A*_0_ (red) versus initial interfacial tension, γ_0_, or the initial interfacial concentration, Γ_0_, observed after the assembly time, *t*; $\widetilde{\gamma }$ is the interfacial tension for the lowest tested concentration of **1** (*c* = 0.001 mmol/L) in chloroform/water. **d** The interfacial tension, γ, of a pendant drop at constant volume is initially rate-limited by the diffusion of the molecular surfactant **1** to the interface; below a critical value corresponding to the critical interfacial concentration, Γ_c_ (blue), its evolution accelerates due to solidification and finally converges to the interfacial tension corresponding to full coverage with a carbon nanoskin (red).

As we will demonstrate below, the carbon-rich surfactant **1** self-assembles at the liquid–liquid interface, and its spontaneous conversion into a carbon nanoskin is triggered once the surfactant concentration at the droplet's surface surpasses a critical interfacial concentration. To quantify the dynamics of this conversion, we have simultaneously characterized the shape, volume and pressure of the pendant drop throughout an entire inflation-deflation cycle, using a similar setup to the one of Danov et al.^23^ (“Methods”, Supplementary Figs. 4–5). This cycle is composed of three steps: (i) inflation; (ii) surfactant assembly for a time, *t*, at constant volume and surface area, *A*_0_; and (iii) deflation of the droplet reducing its surface area to *A* (Fig. 2a and Supplementary Fig. 6). During the inflation process, the experimentally determined pressure, *P*_exp_, at the outlet of the needle always follows the predictions of the pressure, *P*_YL_, that can be independently inferred from the experimental drop shape using the Young–Laplace equation^24^,

1 $${P}_{{{{{{\rm{YL}}}}}}}=\frac{2\gamma }{\bar{R}}-\Delta \rho {gH}$$

where $\bar{R}$ is the radius of curvature of the lower portion of the droplet, $\Delta \rho g$ is the specific gravity of chloroform in water, and $H$ corresponds to the total height of the droplet (Supplementary Information, Section 1.1).

During deflation, *P*_exp_ *initially* remains similar to the pressure predicted by an isosurfactant model, *P*_is_ (Fig. 2a, Supplementary Information, Section 1.2). In the isosurfactant model, we assume that the number of surfactant molecules at the drop surface remains constant throughout deflation, such that

2 $${P}_{{{{{{\rm{is}}}}}}}=\frac{{2\gamma }_{{{{{{\rm{is}}}}}}}}{\bar{R}}-\Delta \rho {gH}$$

where

3 $${\gamma }_{{{{{{\rm{is}}}}}}}=\,\bar{\gamma }-\,\frac{{A}_{0}}{A}\left(\bar{\gamma }-{\gamma }_{0}\right)$$

is the adapted surface tension which accounts for the conservation of surfactant molecules at the interface. The isosurfactant model is consistent with a still liquid behavior of the interface, which is further confirmed by the close match between the pressure predictions of the shape-fitting method and the experimental pressure (*P*_exp_). Since the shape-fitting method allows for the surface tension to freely adjust, this method yields a slightly more precise prediction of the drop pressure than the isosurfactant model, where the evolution of the surface tension is restricted by the conservation of surfactant molecules at the drop interface. Upon continued deflation, however, *P*_exp_ and *P*_is_ start to *diverge* from one another, and the experimental drop shape no longer matches the one predicted by the shape-fitting method. These mismatches point to an anisotropic surface stress of the drop, which can be accounted for by the formation of a solid skin at the interface. Indeed, the experimentally determined pressure converges to the pressure prediction of an isoperimetric model, which assumes an inextensible (solid) behavior along the meridional direction of the drop and predicts a pressure

4 $${P}_{{{\mbox{ip}}}}={\Lambda }_{1}-\Delta \rho {gH}$$

where the Lagrange multiplier ${\Lambda }_{1}$ is associated with the imposed volume constraint (Supplementary Information, Section 1.3). Plots of the liquid-to-solid conversion

5 $$\chi =\frac{P-{P}_{{{{{{\rm{ip}}}}}}}}{{P}_{{{{{{\rm{is}}}}}}}-\,{P}_{{{{{{\rm{ip}}}}}}}}$$

as a function of the relative surface-area change, $1-A/{A}_{0}$, show that, with increasing surfactant concentration, *c*, and assembly time, *t*, an ever-smaller relative reduction in area is necessary to affect the transition from a fluid interface formed from a molecular surfactant (*χ* = 0) to a solid skin (*χ* = 1) (Supplementary Fig. 6). We define the onset of this fluid-solid conversion at the critical surface area, ${A}_{{{{{{\rm{c}}}}}}}$, as the intersection between the tangent at the highest slope of *χ* and the *χ* = 0 axis (Fig. 2b). The key parameter governing the mechanical behavior of the drop upon deflation is the surface tension of the drop immediately before deflation, ${\gamma }_{0}$, which is linked to the initial surfactant surface concentration,

6 $${\Gamma }_{0}=(\bar{\,\gamma }-{\gamma }_{0})/K$$

according to the approximation of a simplified Gibbs isothermal law, where *K =* 1.19 (±0.04) × 10^6^ mN m mol^–1^ is the chemical potential of surfactant **1**, which we have determined by fitting the diffusion-controlled regime of the interfacial concentration after measuring the diffusion coefficient by 2D DOSY NMR spectroscopy (Supplementary Fig. 7, Supplementary Information, Section 1.4).

We observe that, across all experiments, the normalized critical surface area ${A}_{{{{{{\rm{c}}}}}}}/{A}_{0}$ increases linearly with ${\Gamma }_{0}$ and eventually reaches 1 at the critical interfacial concentration ${\Gamma }_{{{{{{\rm{c}}}}}}}=$ 4.2 (±0.3) molecules/nm^2^ (Fig. 2c). At ${\Gamma }_{0}\ge {\Gamma }_{{{{{{\rm{c}}}}}}}$, the interface *immediately* deviates from a fluid behavior upon deflation, implying that for ${\Gamma }_{0} \; < \; {\Gamma }_{{{{{{\rm{c}}}}}}}$, the deflation gradually increases the interfacial concentration until the solidification process is triggered at ${\Gamma }_{{{{{{\rm{c}}}}}}}$. Above ${\Gamma }_{{{{{{\rm{c}}}}}}}$, the interfacial concentration is undefined since Gibbs’ isothermal law is no longer valid due to the solidification. However, the visible onset of wrinkling at a normalized surface area ${A}_{{{{{{\rm{w}}}}}}}/{A}_{0}$ continues to increase steeply with increasing initial interfacial tension ${\gamma }_{0}$ and approaches 1 at approximately ${\gamma }_{{{{{{\rm{w}}}}}}}\simeq$ 5 mN/m, which can thus be regarded as the compressibility limit of the solid nanoskin. It is reassuring that this conversion of the fluid molecular surfactant interface to the solid skin upon deflation is consistent with the time-evolution of the interfacial tension at constant volume, i.e., *without* deflation of the drop (Fig. 2d).

We therefore conclude that there is first a diffusion-limited regime where the surface tension decreases to approximately 21 mN/m, which corresponds to the critical interfacial concentration ${\Gamma }_{{{{{{\rm{c}}}}}}}$, before it drops sharply to finally converge around 5 mN/m, which corresponds to the ${\gamma }_{{{{{{\rm{w}}}}}}}$ observed for complete coverage of the interface with an incompressible nanoskin.

### Structure of the carbon nanoskin

**Fig. 3:**
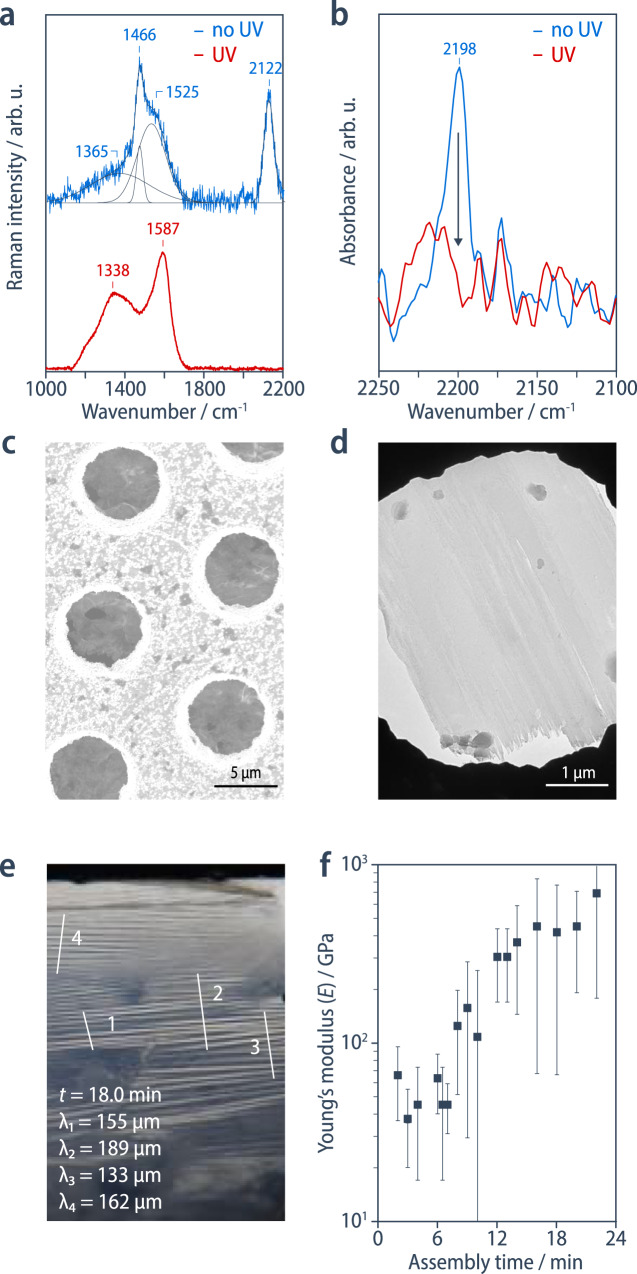
Structural and mechanical characterization of the carbon nanoskin. **a** Raman spectra, and **b** IR reflection-absorption spectra of the carbon nanoskin as-formed and after accelerated carbonization using UV irradiation. **c** Scanning electron micrographs and **d** transmission electron micrographs (SEM, TEM) of the carbon nanoskin after transfer to a copper mesh substrate show a homogeneous and mechanically self-supporting film over many micrometers. **e** Photograph of the nanoskin at a flat chloroform/water interface showing the wrinkling patterns that are formed upon compression and their corresponding wavelength measurement profiles. **f** Conversion of the wavelength to the elastic modulus in bending, considering the thickness determined from the ellipsometry measurements.

An analysis of the chemical and nanoscopic structure of the nanoskin demonstrates that the self-assembled hexayne surfactant **1** rapidly crosslinks into a carbon-rich membrane composed of graphitic flakes and partially reacted carbon–carbon double and triple bonds that continue to evolve into a fully carbonized monolayer (Fig. 1b). UV–vis absorption spectra of the carbon nanoskins, transferred to R-plane sapphire substrates by the Langmuir–Blodgett technique, initially show a broad absorption centered around 768 nm (Supplementary Fig. 8) reminiscent of poly(ene-yne)s but significantly red-shifted compared to typical poly(diacetylene)s^25^. The corresponding Raman spectra of carbon nanoskins transferred to a silicon wafer (Fig. 3a and Supplementary Fig. 9), recorded at an excitation wavelength of 785 nm, exhibit a series of overlapping peaks in the range of 1100–1450 cm^–1^ that can be assigned to carbon–carbon double-bond vibrations, as well as a peak at 2103 cm^–1^ that is characteristic of residual carbon–carbon triple bonds but different from the band observed for the hexayne surfactant **1** at 2084 cm^–1^. These bands are resonance-enhanced because the excitation wavelength lies within the optical absorption of the nanoskin. By contrast, Raman spectra recorded at an excitation wavelength of 532 nm show the D and G bands (1365 and 1525 cm^–1^) characteristic of amorphous carbon nanomaterials, in addition to the carbon–carbon double bond and triple bond vibrations (1466 and 2122 cm^–1^). After aging the sample for 9 weeks at the interface, the carbon–carbon double bond and triple bond vibration bands disappear, suggesting complete carbonization (Supplementary Fig. 9c). When accelerating the process using UV irradiation, one observes similar Raman spectra (Fig. 3a, red curve) showing the D and G bands at 1338 and 1537 cm^–1^, respectively^26^. In the IR reflection-absorption spectrum of the as-formed nanoskin, a band at 2198 cm^–1^  confirms the initial presence of carbon–carbon triple bonds. This band disappears in the fully carbonized material after UV irradiation (Fig. 3b).  Moreover, the significant broadening of the absorption band in the UV–vis spectrum indicates further carbonization of the nanoskin by UV irradiation (Supplementary Fig. 8). The carbonization of surfactant **1** thus proceeds in two concurrent steps, an initial rapid 2D polymerization of the twelve hexayne carbon atoms into cross-linked poly(ene-yne) structures that occurs within seconds to minutes even in the dark, and a parallel but slower graphitization of these structures by cyclization and rearrangement reactions. These processes result in a heterogeneous structure of growing carbon nanomaterial domains separated by less cross-linked poly(ene-yne) structures.

Based on the D and G band positions and the intensity ratio *I*_D_/*I*_G_ (0.6), the final carbon structure can be described as a 2D amorphous carbon containing clusters of sp^2^-hybridized carbon atoms as well as a 10–20% fraction of sp^3^-hybridized carbon atoms that can be attributed to those carbons to which the functional groups remain attached^16^. In this context, it is important to note that the critical interfacial concentration ${\Gamma }_{{{{{{\rm{c}}}}}}}$, at which the carbonization is triggered, corresponds to a mean molecular area of about 24 Å^2^. This value is both consistent with the area required for a dense packing of the phosphonate head groups^27^, and also with the average size of 24–30 Å^2^ of the different “C_12_ carbon tiles” that constitute the carbon layer (Fig. 1b). This geometric consideration can be regarded as a 2D analogy of the 1D molecular packing requirements for the topochemical diacetylene polymerization^28^, and provides a plausible explanation for why the carbonization is triggered mechanically in the case of **1** and then proceeds with a very low activation barrier, whereas it does not occur at all for similar hexayne surfactants with a different head group size (Supplementary Fig. 2b).

Scanning and transmission electron microscopy (SEM, TEM) images of samples transferred to TEM copper grids show continuous films that are self-supporting over many micrometers (Fig. 3d, e). They appear to be homogenous and do not show major defects, except for some wrinkling and draping towards their edges. According to ellipsometry, the carbon nanoskin has a thickness of 3.8 (±1.2) nm, which corresponds to the value expected from the molecular length and remains constant within the error of determination over assembly time (Supplementary Fig. 11c). This assessment was confirmed independently by AFM, giving values of 5.2 (±1.5) nm (*t* = 3 min) that slowly appears to increase with assembly time to approximately 8.7 (±2.8) nm (*t* = 30 min), which we attribute to the transfer of the increasingly rigid nanoskin to the solid substrate (Supplementary Fig. 10, Fig. 11c, and Supplementary Table 1).

### Mechanical properties of the carbon nanoskin

The elastic modulus measured in bending, *E*_*b*_, of the carbon nanoskin can be estimated from the elasto-gravitational wavelength of wrinkles, *λ*^29,30^, upon compression of the nanoskin formed at a flat chloroform/water interface after different assembly times, taking into account its thickness, *h*, of 3.8 nm according to ellipsometry (Fig. 3e, f and Supplementary Fig. 11):

7 $${E}_{b}=\,\frac{12}{{h}^{3}}{\left(\frac{\lambda }{2\pi }\right)}^{4}\rho g$$

**Fig. 4:**
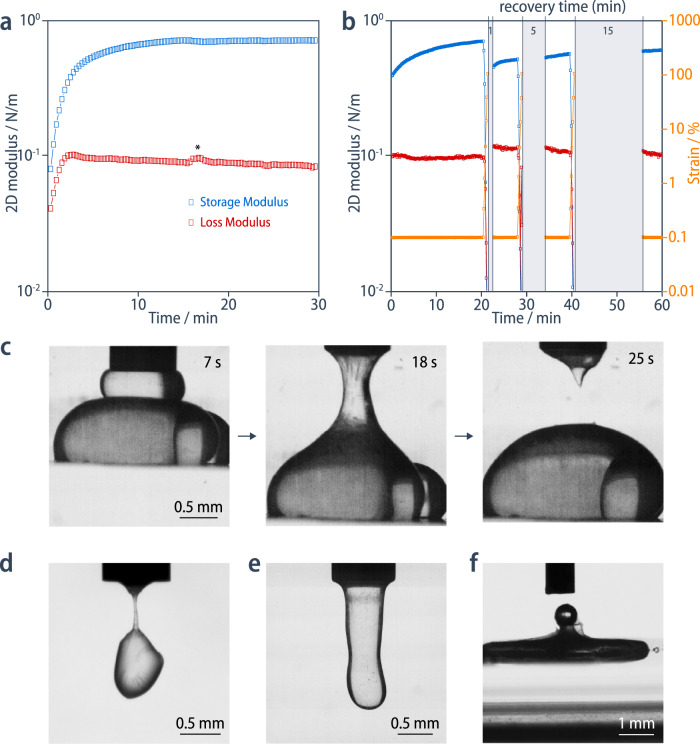
Self-healing and reshaping of the nanoskin. **a** Interfacial rheology shows the evolution of the storage and loss moduli with the formation of the nanoskin (the asterisk indicates an experimental artefact). **b** The self-healing behavior of the nanoskin is followed by interfacial rheology. The nanoskin is ruptured upon a large increase in strain and is left to recover by applying a zero strain; the recovery increases with longer healing time (1, 5, 15 min). **c** A time-sequence of snapshots showing the initial resistance of two droplets to coalescence; subsequent coalescence under compression leads to the creation of a channel between the needle and the droplet; and the breakage of the channel upon withdrawal of the needle (Supplementary Movie 5). **d** Irregularly shaped droplet suspended by a channel with an extremely narrow cross-section. **e** Droplet with an elongated tubular shape. **f** Collapsed droplet containing a gas bubble, which gets trapped by the nanoskin.

This modulus is about 40 GPa after the initial solidification, and it increases with assembly time (over minutes), eventually reaching values as high as 600 GPa, which is of the same order of magnitude of Young’s modulus values reported for other carbon nanomaterials in a series of recent studies (Supplementary Table 2)^31,32–34^. Furthermore, the membrane’s tensile strength, estimated from the tangential forces applied to the membrane necessary to rupture it with a cantilever beam, plateaus at 15 mN/m (Supplementary Fig. 12), corresponding to a stress at rupture of ${\sigma }_{y}\approx 4$ MPa. We attribute the comparably brittle behavior of the carbon nanoskin to its anisotropic and heterogeneous nature as well as the presence of defects, which results in premature failure in the elastic regime. Nevertheless, it is mechanically stable enough to hold the macroscopic weight of a suspended chloroform droplet (Fig. 4d).

Interfacial rheology measurements provide evidence for the elastic behavior of the nanoskin (loss factor tan δ = *G*′′/*G*′= 0.1) from the very early stages (*t* < 2 min, *c* = 0.05 mmol/L) of its formation (Fig. 4a). On one hand, the 2D loss modulus, *G*′, reaches a plateau of 0.085 N/m already after 2 min and slowly decreases over time, demonstrating a progressive decrease in viscous behavior. On the other hand, the 2D storage modulus, *G*′, continues to increase until it converges to 0.75 N/m *(t* > 15 min), a value comparable to other solid interfaces that provides further evidence for the continuing solidification of the carbon nanoskin^31^. While this stationary value of *G*′ would imply a significantly lower 3D elastic modulus than the range determined from the wrinkling experiment, it is important to note that the former is measured in shear as opposed to bending deformation in the latter case. We hypothesize that the difference between the elastic moduli in shear and bending can be attributed to the anisotropic nature of the carbon monolayer densely covered with substituents in a liquid-like state. Similar anisotropic mechanical behavior has been reported recently for the bending deformation of a macroscopic stack of elastic plates interacting through interfacial friction^35^. Finally, the viscous behavior is more predominant at lower concentrations (*t* > 60 min, *c* = 0.02 mmol/L) as evidenced by the higher loss factor (tan δ = 0.6), and overall lower 2D storage and loss moduli with values of 0.011 and 0.0066 N/m, respectively (Supplementary Fig. 13).

### Reshaping and self-healing

The rheological assessment further reveals the unique capability of the carbon nanoskin to self-heal after rupturing the membrane through a substantial increase in strain (0.1–100%). The recovery in modulus increases with healing time and eventually reaches 85% (*c* = 0.05 mmol/L) of the original value after 15 min, which demonstrates that we observe a healing process and are not probing the jamming of flakes (Fig. 4b). Moreover, this behavior is also present at lower concentration (*c* = 0.02 mmol/L), in which case repeated rupture and healing cycles even result in a progressive increase in storage modulus to up to 120% of its original value (Supplementary Fig. 13c). We attribute the self-healing to the crosslinking of the remaining poly(ene-yne) structures at the boundaries between amorphous carbon domains, so that the progressive increase in modulus is consistent with an increase in overall crosslinking density with respect to the original nanoskin.

The capability of the carbon nanoskin to self-heal even allows it to be reversibly reshaped under mechanical stimuli. For example, nanoskin-covered droplets do not readily coalesce when brought in contact, unless the two droplets are compressed together (Fig. 4c, Supplementary Fig. 14, and Supplementary Movies 3–5). This forced coalescence allows the droplet to be reshaped, in a fashion similar to morphogenesis in mitochondria building complex shapes by fusion and fission^36^. For instance, the fusion and fission of two droplets creates conical droplets, or a channel that can subsequently be used for the transfer of fresh solution from one drop to another. The formation of a conical droplet can be reversed by fusion with fresh droplets to restore the liquid behavior of the interface. Other unusual structures such as pendant tubules or suspended droplets can be obtained, while the nanoskin also has the ability to trap gas bubbles (Fig. 4d–f, Supplementary Fig. 14, and Supplementary Movies 6–7).

In conclusion, we have demonstrated that a mechanically stable carbon nanoskin forms spontaneously at the oil–water interface in ambient conditions, where the oil phase is a solution of a reactive carbon-rich surfactant **1**. Due to their amphiphilic nature, the surfactant molecules diffuse to the interface until a critical interfacial concentration is surpassed that triggers the rapid solidification of the interface and eventual conversion of the surfactant molecules into a carbon nanoskin. This carbon nanoskin contains an amorphous and defect-rich carbon monolayer decorated with dodecyl and phosphonatobutyl substituents, respectively, on each side, resulting in an overall thickness of a few nanometers that corresponds to the thickness of the initial self-assembled surfactant monolayer. The carbon nanoskin exhibits elastic moduli reaching more than 40–100 GPa in bending deformation, which is comparable to typical 2D carbon nanomaterials, although the material is comparably brittle. Yet, the spontaneous carbonization at ambient conditions and remaining reactivity of the nanoskin give it the ability to self-heal and be reshaped under mechanical stimuli. Such properties are unprecedented for carbon nanomaterials and are reminiscent of biological membranes, even though they certainly rely on different mechanisms. Based on this unusual combination of properties we envisage such materials to be highly interesting for liquid-in-liquid 3D printing^12^, for advanced manufacturing of microstructured composites using microfluidics^10^, for the formation of “armored” synthetic cells^37^ with increased mechanical stability, and for the biomimetic compartmentalization of catalytic systems^38^ with a rigid but dynamic carbon membrane.

## Materials and methods

### Materials

Octadecyl phosphonic acid was purchased from TCI Chemicals. HPLC-grade chloroform stabilized with ethanol was purchased from Biosolve and used without further purification. Millipore water was obtained from Direct-Q 3 Millipore (Merck, Germany). Surfactant **1** was prepared according to previously published procedure^18^.

### IFT measurements

The interfacial tension was measured using the pendant-drop method on a drop shape analyser (Kruss, DSA30, Germany). A drop of a freshly filtered (PTFE syringe filter, pore size 0.45 µm) solution of **1** in CHCl_3_ was formed in Millipore water and the interfacial tension was measured over a period of 180 s. Supplementary Movie 6 was recorded on this setup.

### DLS measurements

Dynamic light scattering experiments were carried out on a Zetasizer Nano ZS (Malvern Pananalytical, UK). No trace of aggregates was observed in the freshly filtered (PTFE syringe filter, pore size 0.45 µm) solution of **1** in CHCl_3_ (*c* = 0.05 mmol/L).

### Pressure-volume measurements

The pressure inside the drop was determined using a Honeywell Trustability® Board Mount Pressure Sensor (Model: SMT RR). This sensor allows for time-continuous pressure measurements, acquired using a Labview script (Supplementary Fig. 4). We have accounted for the hydrostatic offset between the pressure measured at the location of the pressure sensor and the relevant pressure at the outlet of the blunt injection needle. To do so, we calibrated the sensor using the pressure value inferred from the optical tensiometry analysis during the first stage of drop inflation, where we assume the interface to show unperturbed fluid behavior, that is, isotropic surface stress at the chloroform/water interface ensured by a homogeneous surface tension $\gamma$^39,40^. This assumption for freshly created interfaces is corroborated by the excellent match between the numerical prediction and the experimental data. Moreover, to validate the experimental pressure measurements, we have performed a control experiment by placing the tip of the blunt injection needle at different depths in a chloroform bath and measuring the corresponding pressure (Supplementary Fig. 5). Since the solution inside the syringe and in the bath are the same, there is no capillary pressure difference across the two. We have thus verified the prediction

8 $$P-{P}_{0}={\rho }_{{{{{{\rm{CHC}}}}}}{{{{{{\rm{l}}}}}}}_{3}}g\left(H-{H}_{0}\right),$$

without any fitting parameters and taking ${\rho }_{{{{{{\rm{CHC}}}}}}{{{{{{\rm{l}}}}}}}_{3}}=1490$ kg/m^3^ and $g=$ 9.81 m/s^2^. The pressure–volume curves were measured for freshly filtered chloroform solutions of different volumetric concentrations of the surfactant **1** (*c* = 0.01, 0.02, and 0.05 mmol/L) and for different assembly times, *t*, ranging from a few seconds to a dozen of minutes. A complete description of the physico-chemical methods used to analyze the data is provided in Section 1 of the Supplementary Information.

### Video setup

Supplementary Movies 1–5 and 7 were recorded using a Basler scientific camera (Model: ace acA2040-90uc), mounted with a Navitar lens (6.5× zoom). The drops were generated using a 1 mL syringe, actuated with a New-Era syringe pump (NE-1000) in a quartz cuvette filled with Millipore water. The camera and the pressure sensor (SMT RR 001KD) were synchronized using an in-house developed Labview Code (NI Labview 2017 SP1). All movies and images are reported for *c* = 0.05 mmol/L.

### Substrates and nanoskin preparation

R-plane sapphire and polished silicon wafers were used as substrates for carbon nanoskin transfer. The substrates were cleaned thoroughly by sonication in isopropyl alcohol at room temperature for 10 min before oxygen plasma activation of the surface. The nanoskin was prepared in all cases at the chloroform/water interface from a chloroform solution of surfactant **1** with a concentration of 0.05 mmol/L.

### UV Irradiation

UV irradiation was carried out using of a 250 W Ga‐doped low‐pressure Hg lamp (UV-Light Technology, Birmingham, United Kingdom). The carbonization of the nanoskin was performed at the liquid–liquid interface by irradiation for 1 h in ambient conditions. The lamp was carefully placed 30 cm above the interface which was created in a vial with no lid.

### Raman spectroscopy

The carbon nanoskins were transferred to silicon wafers by the Langmuir–Schaefer technique. Raman spectra were recorded on an inVia™ confocal Raman microscope equipped with lasers of wavelengths 532 and 785 nm.

### IR spectroscopy

A non-UV-irradiated and a UV-irradiated nanoskin were transferred to silicon wafers coated with 200 nm of gold by the Langmuir–Schaefer technique. The infrared reflection-absorption spectra of the nanoskins were measured with with a Fourier transform infrared (FT-IR) spectrometer (Bruker Vertex 80v) equipped with an infrared microscope (Bruker Hyperion 3000).

### UV–vis spectroscopy

The carbon nanoskins were transferred to R-plane sapphire substrates by the Langmuir–Schaefer technique. UV/vis-NIR absorption spectra were recorded on a UV-visible spectrometer (JASCO V-670) using a clean sapphire substrate as a reference.

### Sample preparation for AFM and ellipsometry

The AFM and ellipsometry samples were prepared by transferring the non-irradiated carbon nanoskins to silicon wafers using the Langmuir–Blodgett technique. The silicon wafer substrate was placed in the chloroform subphase before creation of the interface by layering of Millipore water. The substrate was withdrawn using a dip-coater (KSV DC) after various waiting time (3, 10, 30 min) at a speed of 75 mm/min.

### Atomic force microscopy (AFM)

AFM measurements were performed in tapping mode using an Asylum Research Cypher S scanning probe microscope in ambient air to obtain topographical images from the height signal. All scans were performed at 512 lines resolution using standard aluminum back-side coated silicon cantilevers purchased from Mikromasch (HQ:NSC18/Al BS, resonant frequency 75 kHz, spring constant 2.8 N m^–1^, probe tip radius ca. 8 nm). From these height signal images, we attempted to determine the nanoskin thickness. A region of interest was selected from each image, (in red Supplementary Fig. 10a–c) that did not contain any severe artifacts from the height signal. Image leveling was then performed on the region of interest using *Gwyddion* software. Histographic analysis of the leveled image (bin size of 0.1 nm) shows two prominent peaks: one corresponding to the substrate height centered at ca. 0 nm, another corresponding to the nanoskin thickness. Nevertheless, imperfections in the leveling process and the presence of unavoidable artifacts (such as particles or wrinkles) would contribute to smaller peaks hidden within the height distribution. The histograms were deconvoluted using *OriginPro* software by fitting all the deconvoluted peaks to a Gaussian distribution function. From the fitting results, the center of gravity and full width at half maximum (FWHM) values were obtained for the two deconvoluted peaks that contribute to the substrate height and nanoskin height. The mean thickness of the nanoskin was determined as the difference in the center of gravity values of both peaks, whereas the error was determined as half of the sum of FWHM values of both peaks (Supplementary Table 1).

### Ellipsometry

The ellipsometry measurements were performed on a variable angle spectroscopic ellipsometer from Semilab ZRt (SE2000). Ellipsometric data were recorded at an incidence angle of 65°, 70° and 75° and a wavelength range of 245–990 nm at 5 different locations for every sample. The ellipsometric data, consisting of the angles Ψ and Δ, were analyzed using the software provided with the instrument (Spectroscopic Ellipsometry Analyzer v1.6.1, Semilab). The calculation method was based on a three-layer model (silicon, silicon oxide, nanoskin) with the refractive index of the nanoskin set to 1.45.

### Electron microscopy

The SEM and TEM samples were prepared by transferring the non-irradiated carbon nanoskins to TEM copper grids with 2000 l/inch circular mesh by a Langmuir–Blodgett technique. The substrates were placed in the chloroform subphase before creation of the interface by layering of Millipore water. The substrates were withdrawn using a dip-coater (KSV DC) at a speed of 75 mm/min.

### Transmission electron microscopy (TEM)

TEM images were recorded on a Thermo Scientific TM TALOS F200X operating with an accelerating voltage of 200 kV. Bright-field mode was used for high-resolution transmission electron microscopy (HR-TEM). To avoid nanoskin damage induced by the electron beam, the spot size was increased only when areas of interest in the specimen were selected for image recording. Images were acquired on a CETA FEI camera.

### Scanning electron microscopy (SEM)

Bright-field SEM images were recorded on a Zeiss Gemini SEM300 electron microscope operated in high vacuum (HV) mode. Electrons were collected by a standard Everhart-Thornley secondary-electron (SE) detector to observe topological details of the nanosheets and by a Gemini II column with an in-lens detector to obtain high contrast. A 5 mm working distance and an acceleration voltage of 3 keV were used. The lens aperture of 30 µm was chosen small enough to avoid spherical aberrations.

### 2D DOSY NMR spectroscopy

The determination of the diffusion coefficient of surfactant **1** was performed by 2D DOSY NMR spectroscopy in a mixture of CDCl_3_/CD_3_OD (4:1) to increase solubility of **1** and was carried out on a Bruker Avance III 400 spectrometer at frequencies of 400.13 MHz for ^1^H nuclei.

### Determination of the Young’s modulus

The interface of solution **1** (*c* = 0.05 mmol/L in chloroform) and water was created in a glass cuvette of dimensions 25 × 50 × 10 mm^3^ (width × length × depth). The interface was then compressed after different assembly times, *t*, using an aluminum blade of width 24 mm, brought in contact with the chloroform/water interface and then slowly translated in the long direction of the cuvette. The resulting wrinkling pattern was then photographed using a camera (Nikon D850) with a macro lens. For each image, four average wrinkling wavelengths were measured in four different regions of the membrane (each one of these 4 values were obtained by averaging over approximately 10 wavelengths, Supplementary Fig. 11). The wavelengths were used to estimate the Young’s modulus using the relation in Eq. (7) of the main text. The thickness used was 3.8 nm as determined by ellipsometry.

### Tensile testing

A short portion of an elastic beam (length $l$) was permanently bent to be straight and perpendicular to the main part of the beam (length $L$). The straight end of the beam was clamped to a rigid support pointing downwards and the short horizontal portion (length $l$) was brought in contact with a flat interface of solution **1** (*c* = 0.05 mmol/L in chloroform) and water. The container was then moved in the direction perpendicular to the horizontal portion of the beam at an imposed speed of *v* = 20 ± 2 mm/min (Supplementary Fig. 12). The deflection of the cantilever beam could be translated into the bending force *F* exerted by the carbon nanoskin, by *F* = *kx*, where *x* is the displacement of the cantilever and *k* is its spring constant. At a given maximum displacement *x*, the membrane ruptures and the cantilever jumps to a configuration close to its original one (nearly straight), and the maximum force was recorded. The maximum tensile strength in mN/m sustained by the membrane was then calculated by dividing the maximum force by the horizontal length *l*. Tensile tests were performed for different assembly times, *t*.

### Interfacial shear rheology

Interfacial rheology experiments were performed in ambient conditions, at the chloroform/water interface (*c* = 0.01–0.05 mmol/L), a Teflon trough using a double-wall ring geometry (DWR) mounted on a Discovery Hybrid Rheometer 3 (TA Insruments, USA). The time sweep was performed at a frequency *ω* = 1 rad/s and a strain *γ* = 0.1%. The self-healing experiment was performed in multiple steps at a constant frequency *ω* = 1 rad/s; (i) the formation of the nanoskin was monitored at a constant strain *γ* = 0.1% for 20 min; (ii) the nanoskin was ruptured by ramping the strain from 0.1 to 100% in 1 min; (iii) a healing time of 1 min was observed during which no measurement was done; (iv) the nanoskin recovery was monitored for 5 min at a strain of *γ* = 0.1%; (v) steps (ii)–(iv) were repeated two more times with increasing healing times of 5 and 10 min in step (iii).

## Supplementary information

Supplementary Information

Description of Additional Supplementary Files

Supplementary Movie 1

Supplementary Movie 2

Supplementary Movie 3

Supplementary Movie 4

Supplementary Movie 5

Supplementary Movie 6

Supplementary Movie 7

## Data Availability

The datasets generated during and/or analysed during the current study are available from the corresponding author on request.
