# Supplementary material for: Spontaneous formation of a self-healing carbon nanoskin at the liquid–liquid interface
Source: Nat Commun. 2022 Aug 23;13:4950. doi: 10.1038/s41467-022-31277-5 (PMC9399178; doi:10.1038/s41467-022-31277-5)
Supplement: Supplementary file 1 — Supplementary Information [file 41467_2022_31277_MOESM1_ESM.pdf]

Spontaneous Formation of a Self-Healing Carbon Nanoskin at the Liquid-Liquid Interface

Supplementary Information

Enzo Bomal,[1] Paul Grandgeorge,[2] Reuben Yeo,[1] Nicolas Candau,[1] Pedro Reis,[2] Holger Frauenrath[1]*

[1] Ecole Polytechnique Fédérale de Lausanne (EPFL)
Institute of Materials
Laboratory of Macromolecular and Organic Materials

EPFL–STI–IMX–LMOM
MXG 037, Station 12
1015 Lausanne, Switzerland

holger.frauenrath@epfl.ch

[2] Ecole Polytechnique Fédérale de Lausanne (EPFL)
Institute of Mechanical Engineering
Flexible Structures Laboratory

Table of Contents

1. Physicochemical Models **2**

1.1 Pendant Drop and Interfacial Tension Measurements using the Young-Laplace equation 2

1.2 Iso-Surfactant Model 3

1.3 Iso-Perimetric Model 5

1.4 Time-Evolution of Interfacial Tension 7

2. Supplementary Figures **100**

3. Supplementary Tables **244**

1. Physicochemical Models

1.1 Pendant Drop and Interfacial Tension Measurements using the Young-Laplace equation

We determine the interfacial tension of a liquid drop using the pendant-drop method (see Experimental Section and Supplementary Fig. 5).[34] We apply the well-established Young-Laplace equation for a liquid drop hanging from a vertical blunt needle (capillary) of diameter D.[24] The drop has a specific weight $\Delta\rho g$ ($\Delta\rho = 490$ kg/m^3 in our case) and an interfacial tension γ. We define the drop shape by its axisymmetric surface given by the profile $R(Z)$.

The pressure jump at a curved interface follows the relation:

$$\Delta P(Z) = (P_{\mathrm{int}} - P_{\mathrm{ext}}) = \gamma\left(\frac{1}{R_1(Z)} + \frac{1}{R_2(Z)}\right), \tag{1}$$

where R_1 and R_2 correspond to the principal radii of curvature of the interface. Moreover, hydrostatic equilibrium yields:

$$\Delta P(Z) = \Delta P_0 - \Delta\rho g Z \tag{2}$$

where ΔP_0 is the pressure jump at $Z = 0$ (at the bottom of the drop). We introduce the arclength variable S along the drop profile ($S = 0$ corresponds to the bottom of the drop) and the angle $\theta(S)$ between the tangent vector of the profile and the horizontal axis. The principal radii of curvature are then written:

$$\frac{1}{R_1} = \frac{d\theta}{dS} \tag{3}$$

and

$$\frac{1}{R_2} = \frac{\sin\theta}{R}. \tag{4}$$

Noting that $R_1(S = 0) = R_2(S = 0) = \bar{R}$ (where $\bar{R}$ is unknown), we obtain the following system of ordinary differential equations (ODEs) (equations 5–7):

$$\frac{d\theta}{dS} = -\frac{\sin\theta}{r} + \frac{2}{\bar{R}} - \frac{\Delta\rho g Z}{\gamma}, \tag{5}$$

$$\frac{dR}{ds} = \cos\theta, \tag{6}$$

$$\frac{dZ}{dS} = \sin\theta, \tag{7}$$

with the boundary conditions $R(S=0) = Z(S=0) = \theta(S=0) = 0$.

Assuming that the surface tension γ of the drop is known, the above system of ODEs comprises two unknowns, the radius of curvature $\overline{R}$ at the bottom of the drop and the total arclength of the drop profile S_f. The additional equations required to determine $\overline{R}$ and S_f are the geometrical constraints:

$$2R(S_f) = D \tag{8}$$

and

$$Z(S_f) = H \tag{9}$$

where H is the total height of the drop.

To determine the surface tension of a pendant drop, the value of γ is optimized for the computed theoretical profile computed with this model such that the best fit is obtained for the experimental profile of the drop. For this, we first convert the photographs of the drop recorded by the camera into binary images, from which we then extract the experimental profile $R_{\mathrm{exp}}(Z_{\mathrm{exp}})$ using an edge-detection image processing algorithm, developed in-house using MATLAB. Note that the experimentally measured distance from the drop apex to any height along the drop Z_{exp} is defined discretely by the pixels size. Finally, the optimal value of γ is determined by minimizing the target error function $\sum_{i=1}^{M} |R(Z(S_i)) - R_{\mathrm{exp}}(Z_{\mathrm{exp}-i})|$, where i corresponds to the height in pixels and the values of S_i are taken so as to have $Z(S_i) = Z_{\mathrm{exp}-i}$. The total height of the drop in pixels is M. The validity of this approach is demonstrated in Supplementary Fig. 4, where we superimpose the experimental image of the drop and the corresponding theoretically predicted profile obtained using the model described in this section.

Once the best-fit value of the surface tension is obtained, we then infer the value of the pressure at the outlet of the capillary as:

$$P(Z = H) = \frac{2\gamma}{\overline{R}} - \Delta\rho g H. \tag{10}$$

This value is then taken as the inferred pressure of the droplet P_{YL}

1.2 Iso-Surfactant Model

The iso-surfactant model describes the behavior of a drop covered with a molecular surfactant upon deflation. It assumes that the adsorption and desorption kinetics of surfactant molecules at the interface

are significantly slower than the typical time scales at which interface surface areas change (the drop is typically deflated in less than 2 s). Therefore, we consider that the number of surfactant molecules N (in moles) remains constant at the surface of the drop throughout its deflation. Consequently, the interfacial tension of the drop is not constant throughout its deflation. We consider that the interfacial tension follows the linear law (simplified Gibbs isothermal):

$$\gamma = \bar{\gamma} - K\,\Gamma, \tag{11}$$

where $\bar{\gamma}$ is the interfacial tension of the pure solvent (*i.e.*, without surfactant; $\bar{\gamma} \approx 30$ mN/m for the chloroform/water interface), K is the chemical potential expressed in Nm/mol and Γ is the surfactant surface concentration in mol/m^2 at the interface. The interfacial tension at the state 0 (right before deflation is initiated) is therefore given by

$$\gamma_0 = \bar{\gamma} - K\,\Gamma_0. \tag{12}$$

In addition, since the number of surfactant molecules N at the interface is assumed to remain constant, we have

$$\begin{aligned} N(t) &= N_0 \\ &= A_0\Gamma_0 \\ &= A(t)\cdot\Gamma(t), \end{aligned} \tag{13}$$

where A_0 and $A(t)$ are, respectively, the surface area of the drop at state 0 and at a time t. Finally, rewriting Γ_0 as a function of A_0, $A(t)$, and γ_0, the relation between the interfacial tension of the drop $\gamma(t)$ and its surface area at time t becomes:

$$\gamma(t) = \bar{\gamma} - \frac{A_0}{A(t)}(\bar{\gamma} - \gamma_0). \tag{14}$$

To obtain the pressure predicted by this iso-surfactant model upon drop deflation, we initialize the shape of the drop with its volume right before deflation V_0, at the (known) surface tension γ_0. This allows for the prediction of the initial drop surface area A_0. This initial state corresponds to the step $k = 0$ of the numerical computation. The drop volume is then iteratively decreased, numerically, and the interfacial tension γ is updated at each step k using equation (15), adapted as:

$$\gamma_k \simeq \bar{\gamma} - \frac{A_0}{A_{k-1}}(\bar{\gamma} - \gamma_0). \tag{15}$$

which allows to compute the equilibrium shape at each step. Imposing the volume $V_k = V_0 - k\,\Delta V$ of the drop which has a surface tension γ_k allows us to obtain the pressure P_{is} predicted by the iso-surfactant model (ΔV is the incremental reduction of volume between each iteration step)

$$P_{\text{is}} = \frac{2\gamma_{\text{is}}}{\bar{R}} - \Delta\rho g H. \tag{16}$$

The iterative computational procedure described above was performed for each experiment using MATLAB. Moreover, a sensitivity analysis showed that our results were independent of the chosen value of the volume step size, ΔV.

1.3 Iso-Perimetric Model

For the iso-perimetric model, we consider that the meridional outer perimeter described by the perimeter of the projection of the drop remains constant. This assumption is motivated by the solid-like behavior of the skin enveloping the drop. We assume that this skin allows effective shrinking under compression through wrinkling in the hoop (or *circumferential*) direction of the drop, but no effective length increase through stretching under tension along the meridional direction. The asymmetry of the response (no stiffness in compression and no extension under tension) is commonly observed in solid slender structures. It is therefore important to highlight that this model does not assume a liquid behavior of the interface (*i.e.*, drop surface stresses are not isotropic). Moreover, since this model assumes global inextensibility in the meridional direction, we do not account for any elastic stretching energy of the skin. To obtain the governing equations related to this problem, we write the total energy $\mathcal{U}$ of the drop:

$$\mathcal{U} = \int_0^H \rho g Z\, \pi R^2(Z)\, \mathrm{d}Z \; - \Lambda_1 \int_0^H \pi R^2(Z)\, \mathrm{d}Z - \Lambda_2 \int_0^H \sqrt{1 + R'^2(Z)}\, \mathrm{d}Z. \tag{17}$$

The first term in equation (17) corresponds to the gravitational energy of the drop, Λ_1 (force per unit area) is the Lagrange multiplier associated with the imposed volume of the drop and Λ_2 (force) is the Lagrange multiplier associated with the fixed meridional perimeter of the drop projection. Note that $Z = 0$ corresponds to the bottom part of the drop whereas $Z = H$ is the height at which the drop is in contact with the blunt capillary needle. The Lagrangian is then given by:

$$\mathcal{L} = \rho g Z \pi R^2(Z) - \Lambda_1 \pi R^2(Z) - \Lambda_2 \sqrt{1 + R'^2(Z)}\,. \tag{18}$$

To simplify the problem, we nondimensionalize the relevant variables as follows:

$$r = \frac{R}{R_0}, \quad z = \frac{Z}{R_0}, \quad h = \frac{H}{R_0}, \quad \lambda_1 = \frac{\Lambda_1}{\Delta\rho g R_0}, \quad \lambda_2 = \frac{\Lambda_2}{\pi\Delta\rho g R_0^3}, \quad \ell = \frac{\mathcal{L}}{\pi\Delta\rho g R_0^3}$$

where R_0 is the radius of the blunt capillary from which the drop hangs. This leads to the dimensionless Lagrangian:

$$\ell = r^2 z - \lambda_1 r^2 - \lambda_2\sqrt{1 + r'^2} \tag{19}$$

and the system is governed by the equation:

$$\frac{\partial \ell}{\partial z} - \frac{\mathrm{d}}{\mathrm{dz}}\left(\frac{\partial \ell}{\partial r'}\right) = 0 \tag{20}$$

which yields to the second order differential equation for $r(z)$:

$$r''(z) = \frac{2}{\lambda_2}\,(\lambda_1 - z)\ r(z)\ [1 + r'^2(z)]^{3/2} \tag{21}$$

with initial conditions:

$$r(z = 0) = \ 0, \qquad r'(z = 0) = \infty. \tag{22}$$

The Lagrange multipliers λ_1 and λ_2 are obtained, respectively, by imposing a volume V and a meridional perimeter L. The height of the drop H is constrained by an additional boundary condition:

$$r(z = h) = 1, \tag{23}$$

We solve the differential equation (21) using MATLAB with the in-built ode-solver "ode45". The initial condition corresponding to infinity in equation (22) is set to an arbitrarily large number numerically ($1{\cdot}10^4$). The pressure predicted by this iso-perimetric model is given by the pressure at $Z = 0$, that is Λ_1, corrected by the hydrostatic pressure loss:

$$P_{\mathrm{ip}} = \Lambda_1 - \Delta\rho g H. \tag{24}$$

The comparison between the above model and the experimental data is performed by computing P_{ip} for different volumes of a drop, with a fixed meridional perimeter L_0, measured on the experimental drop, right before deflation is initiated. Therefore, this prediction for the pressure corresponds to the ideal case where the drop perimeter would remain constant throughout the deflation. In the experiments, given that the drop displays an initial purely fluid-like behavior upon deflation (described by the iso-surfactant model), the outer meridional perimeter of the drop is observed to first decrease by a finite amount before becoming constant. For this reason, the experimental pressures do not exactly reach the

ones predicted by this model. Nevertheless, the combination of experimental pressure measurement and this predictive model allows us to quantify the conversion of the drop interface from fluid to solid with sufficient accuracy.

1.4 Time-Evolution of Interfacial Tension

Once inflated, the interfacial tension of a pendant drop of surfactant solution changes over time. Indeed, when a fresh interface is generated, the surfactant molecules need time to diffuse to and subsequently adsorb at the interface. We use the following simple model to describe the surface tension of an interface as function of the surfactant surface concentration $\Gamma(t)$ (in mol/m^2) adsorbed onto it:

$$\gamma(t) = \bar{\gamma} - K\,\Gamma(t) \tag{25}$$

where $\bar{\gamma}$ is the interfacial tension of the pure solvent without surfactant in mN/m ($\bar{\gamma} \approx 30$mN/m for the chloroform/water interface), and K is a coefficient expressed in mN·m/mol. This relation suggests that $\gamma = \bar{\gamma}$ when no surfactant molecule is adsorbed at the interface ($\Gamma = 0$) and that it decreases for increasing surfactant surface concentration.

To gain insight into the time evolution of the surface tension, we need to consider the diffusion of surfactant molecules *inside* the drop; *i.e.*, in the bulk. For this, we consider that the radius of curvature is larger than the typical length scale over which diffusion occurs, and therefore restrict our analysis to a one-dimensional model. Then, the relation between local time and space variation of surfactant volumetric concentration $c(x,t)$ (in mol/m^3) is described by Fick's law of diffusion:

$$\frac{\partial c}{\partial t} = D\frac{\partial^2 c}{\partial x^2} \tag{26}$$

where x is the coordinate normal to the droplet surface and D is the surfactant diffusivity in chloroform. In our case, the initial condition is given by $c(x, t = 0) = c_\infty$. Moreover, spatial boundary conditions are $c(x = 0, t) = 0$ and $c(x \to \infty, t) = c_\infty$. This corresponds to a semi-infinite space initially at a homogeneous concentration c_∞. At times $t > 0$, the concentration at $x = 0$ is fixed to 0, thus emulating a sink of infinite capacity, which approximates the interface, and is assumed to adsorb surfactant molecules infinitely fast, considering only the diffusion-limited regime. Note that other more accurate models such as the Langmuir adsorption relation [35] do not assume $c(x = 0, t) = 0$, but instead, relate

$c(x=0,t)$ to the surfactant surface concentration $\Gamma(t)$ at the interface. However, the Langmuir adsorption relation requires the input of additional physico-chemical constants such as the maximal surface concentration and the Langmuir adsorption constant. Since these two constants are not reported in literature for surfactant **1**, and their experimental characterization goes beyond the scope of this study, we turned to the assumption of $c(x=0,t)=0$ in the employed model.

The analytical solution of equation (27) using these boundary conditions reads:

$$c(x,t) = c_\infty \left[1 - \operatorname{erfc}\left(\frac{x}{2\sqrt{Dt}}\right)\right] \tag{27}$$

and the molecular flux J (mol·m^{-2}·s^{-1}) passing through a unit surface is given by:

$$J = -D\frac{\partial c}{\partial x} = -\sqrt{\frac{D}{\pi t}}\, c_\infty \exp\left(-\frac{x^2}{4Dt}\right). \tag{28}$$

In the limit of an interface with infinitely fast adsorption kinetics and no desorption, we write the rate of change of surfactant surface concentration as

$$\dot{\Gamma}(t) = -J(x=0,t) = c_\infty \sqrt{\frac{D}{\pi t}}, \tag{29}$$

which upon integration yields

$$\Gamma(t) = \Gamma_i + \int_0^t \dot{\Gamma}(t)\mathrm{d}\,t \; = \; \Gamma_i + 2\cdot c_\infty \sqrt{\frac{Dt}{\pi}} \tag{30}$$

where $\Gamma_i = \Gamma(t=0)$. Note that we assume that the initial surfactant surface concentration Γ_i can be non-zero without this having an influence on the initial homogeneity of the surfactant volumetric concentration $c(x, t=0) = c_\infty$.

The time dependence of the surface tension $\gamma(t)$ is finally expressed as:

$$\gamma(t) = \bar{\gamma} - K\Gamma_i - 2Kc_\infty \sqrt{\frac{Dt}{\pi}} \; = \gamma_i - \alpha\, c_\infty \sqrt{t} \tag{31}$$

with $\gamma_i = \bar{\gamma} - K\Gamma_i$ as the initial surface tension (at $t = 0$) and $\alpha = 2K\sqrt{D/\pi}$. Experimentally, K is unknown, while D can be estimated by 2D DOSY NMR spectroscopy (Supplementary Fig. 7).

In Supplementary Figure 7, we plot curves for $\Delta\gamma(t) = \gamma_i - \gamma(t)$ obtained from seven experiments at a surfactant volumetric concentration $c_\infty = 0.02$ mM and different assembly times, *t*. The prediction

$$\Delta\gamma(t) = \alpha\, c_\infty \sqrt{t} \tag{32}$$

works well for sufficiently long times ($t > 10$ s) with a fitted coefficient $\alpha = 30 \pm 1$ mN·m^{2}·mol^{-1}·s$^{-1/2}$. For shorter times, the data carries noise, but do not seem to follow the $\sqrt{t}$ prediction. It can be hypothesized that the initial 10 s do not correspond to the diffusion-limited regime, but to a regime where adsorption kinetics also play an important role.

Fitting the parameter α to the data, we find $\alpha = 30$ (±1) mN·m^{2}·mol^{-1}·s$^{-1/2}$, which enables us to calculate K, knowing that $D = 5 \cdot 10^{-10}\ \mathrm{m^2/s}$,

$$K = \frac{\alpha}{2}\sqrt{\frac{\pi}{D}} = 1.19 \pm 0.04 \cdot 10^{6}\ \mathrm{mN \cdot m \cdot mol^{-1}}\,. \tag{33}$$

This value of K is then used to calculate $\Gamma(t)$

$$\Gamma(t) = \frac{\bar{\gamma} - \gamma(t)}{K}. \tag{34}$$

At lower values of the concentration, the behavior was more challenging to detect. At $c_\infty = 0.05$ mmol/L, the process was too fast to allow a fitting of the data. At $c_\infty = 0.01$ mmol/L, the surface tension reaches a plateau value. A possible explanation is the complete depletion of surfactant molecules of the drop. Indeed, we give an estimate for the maximal surface area concentration $\Gamma_{\max}$ attainable for a spherical drop of radius R, volume $V = 4\pi R^3/3$ and surface area $A = 4\pi R^2$, with initial volumetric surfactant concentration c_∞:

$$\begin{aligned} \Gamma_{\max} &= \frac{c_\infty V}{A} \\ &= \frac{c_\infty\, R}{3} \\ &\simeq 3.3 \times 10^{-6}\, \frac{\mathrm{mol}}{\mathrm{m}^2} \\ &\simeq 2\ \mathrm{molecules/nm^2} \end{aligned} \tag{35}$$

for a drop of radius $R = 1$ mm and initial concentration $c_\infty = 0.01$ mM. This shows that around volumetric concentrations of 0.01 mM, the value of the final surfactant surface concentration might be dictated by the lack of spare molecules to adsorb at the interface, rather than by an actual saturation of the interface. Another possible scenario is that the surfactant desorbs from the interface when the bulk concentration is low. The observed plateau surface tension would then be a dynamic equilibrium state between the adsorption and desorption of surfactant molecules at the interface.

2. Supplementary Figures

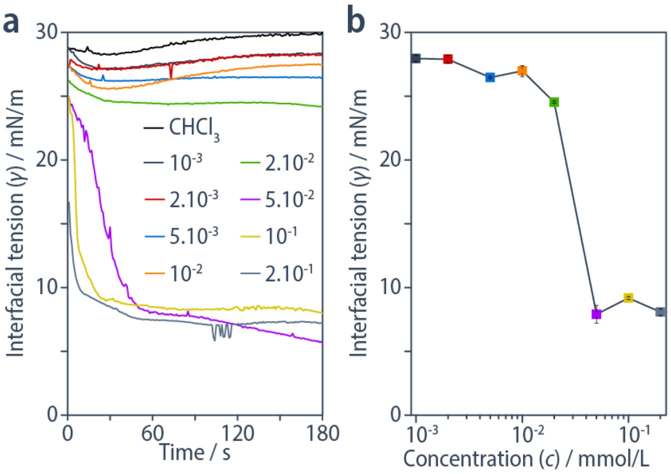

Supplementary Figure 1. Interfacial tension of chloroform solutions of **1** at various concentrations. **a**, The pendant-drop method was used to determine the interfacial tension of chloroform solutions of the carbon-rich surfactant **1** (at different concentrations, c) in deionized water over time. Pure chloroform in water has an interfacial tension of $\bar{\gamma} = 30$ mN/m. **b**, For surfactant solutions at concentrations of $c = 0.001–0.020$ mmol/L, the interfacial tension seems to plateau after tens of seconds at a value slightly lower than the interfacial tension of pure chloroform. For concentrations $c \geq 0.05$ mmol/L, the interfacial tension decreases sharply when the surface tension reaches approximately $\gamma \leq 20$ mN/m and eventually equilibrates at values of $\gamma = 5–10$ mN/m.

a

b

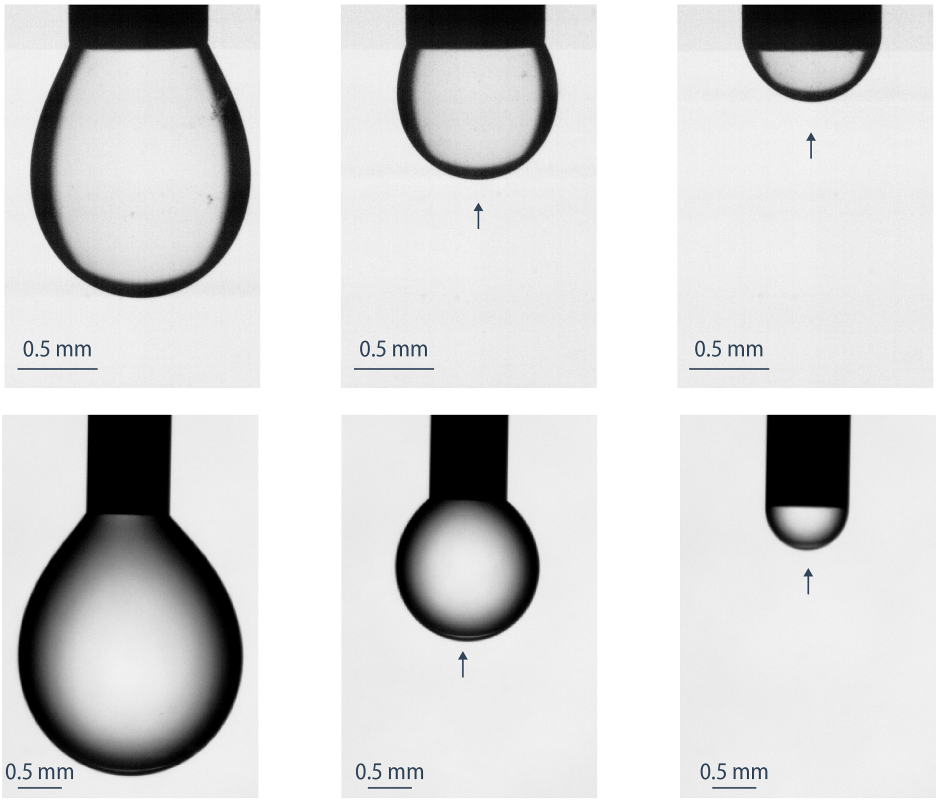

Supplementary Figure 2. Snapshots of control experiments. Upon deflation, a droplet of a chloroform solution of **a**, octadecyl phosphonic acid (c = 0.05 mmol/L) and **b**, octacosa-5,7,9,11,13,15-hexaynoic acid (c = 0.05 mmol/L) maintain an equilibrium drop shape throughout the entire process, which is consistent with the Young-Laplace model and shows no signs of wrinkling.

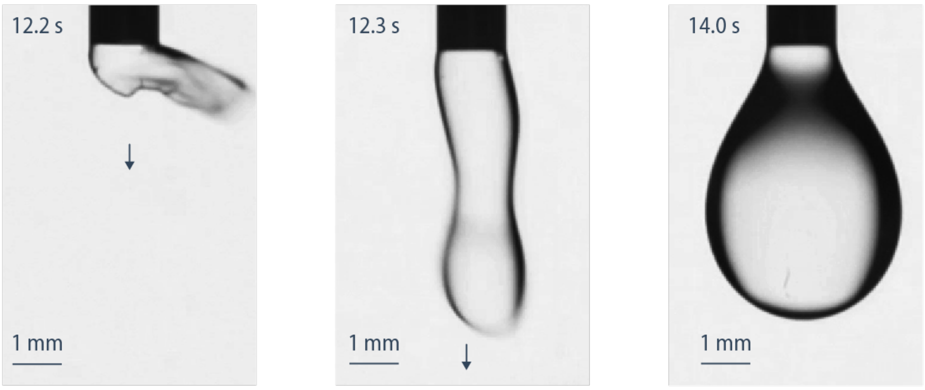

Supplementary Figure 3. Snapshots of Supplementary Movie 2. A droplet of surfactant **1** in chloroform, upon rapid re-inflation, demonstrates that the nanoskin-covered droplet can reversibly return to a stable configuration, again, dominated by surface tension, where the membrane regenerates within a few seconds (Supplementary Movie 2). Arrows indicate inflation or deflation.

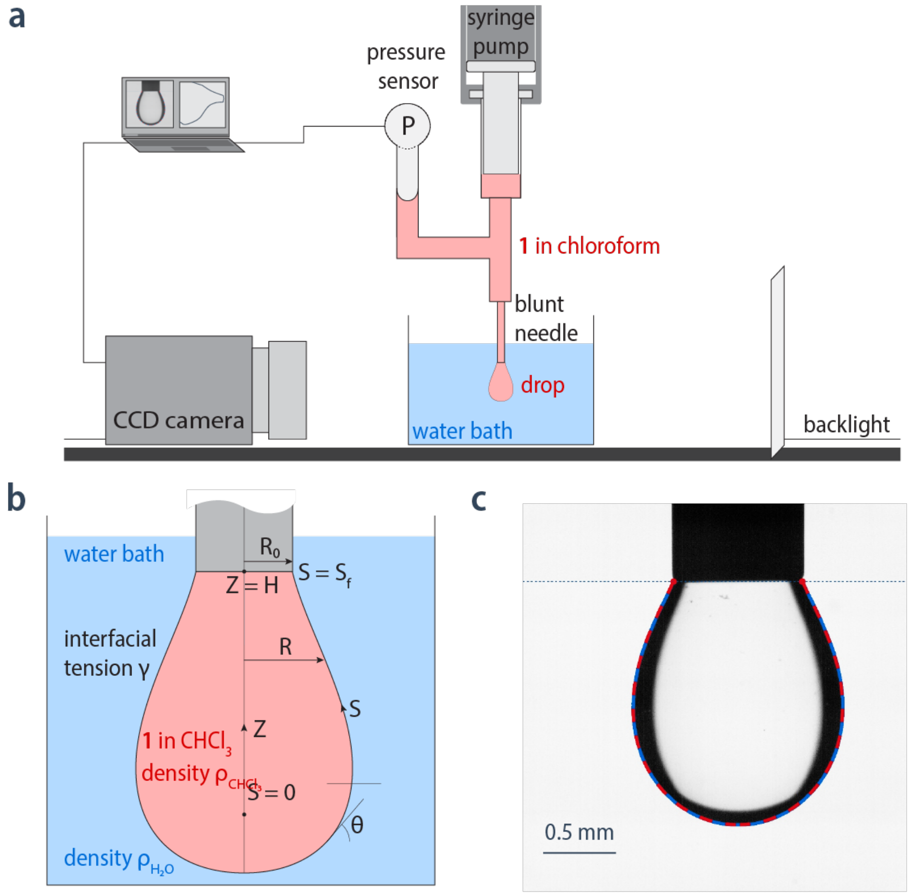

Supplementary Figure 4. Experimental setup for pressure-volume curve measurements. **a**, Schematic diagram of the experimental apparatus used to measure the pressure inside the pendant drop. The hydrostatic connection between the drop and the sensor is realized using a T-junction. The resulting discrepancy between the measured pressure and the real pressure at the outlet of the injection blunt needle is corrected using a synchronized optical tensiometry analysis. **b**, Schematic diagram of the drop and the key physical and geometric parameters. The drop of chloroform solution of 1 hangs from a blunt needle of outer radius R_0. This drop has an interfacial tension γ with the surrounding water and an effective density of $\Delta\rho = \rho_{\mathrm{CHCl_3}} - \rho_{\mathrm{H_20}}$. We describe the shape of the drop using the one-dimensional arclength S as $R(S)$, the radius of the drop around its symmetry axis at location S. The bottom of the drop corresponds to $S = 0$ (height Z=0) and the upper part, in contact with the needle, is located at arclength $S = S_f$, where $Z = H$, the total height of the drop. Finally, to fully describe the geometry of the system, we introduce the angle θ between the tangent of the drop surface along the meridional direction and the horizonal. **c**, Representative example of the image analysis of a freshly generated drop of a chloroform solution of surfactant 1 in a water bath. The blue line corresponds to the drop profile obtained through image processing, and the red dashed line corresponds to the best fitting shape prediction (optimal surface tension: $\gamma = 25.7$ mN/m), both superposed on an experimental photograph. For this example, the diameter of the blunt needle is 0.91mm and volume of the drop is $V = 16$ μL.

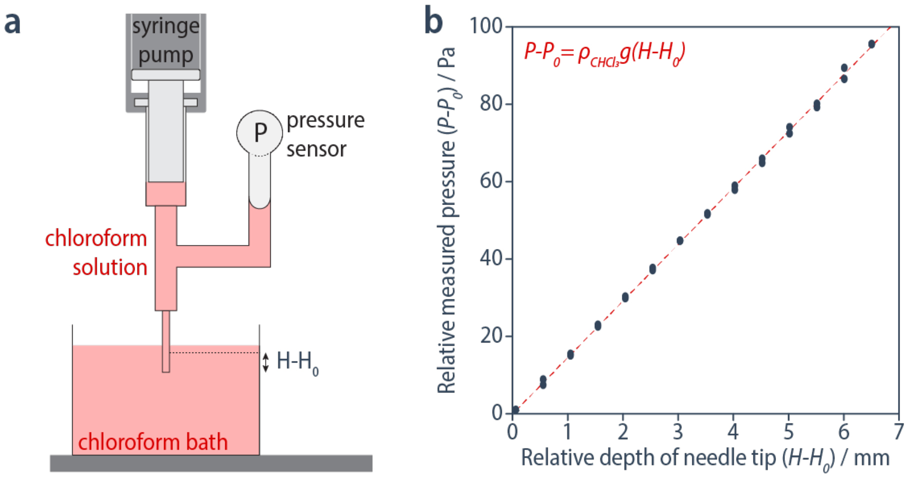

Supplementary Figure 5. Calibration of the setup. **a**, The calibration was verified by performing a hydrostatic pressure measurement: the needle was placed in a chloroform bath at different depths, and the pressure was recorded at each height step. **b**, The dashed line corresponds to the prediction $P - P_0 = \rho_{\mathrm{CHCl_3}}\, g(H - H_0)$ with no fitting parameters, using $\rho_{\mathrm{CHCl_3}} =$ 1490 kg/m^3 and $g = 9.81$ m/s^2 (see Materials and Methods).

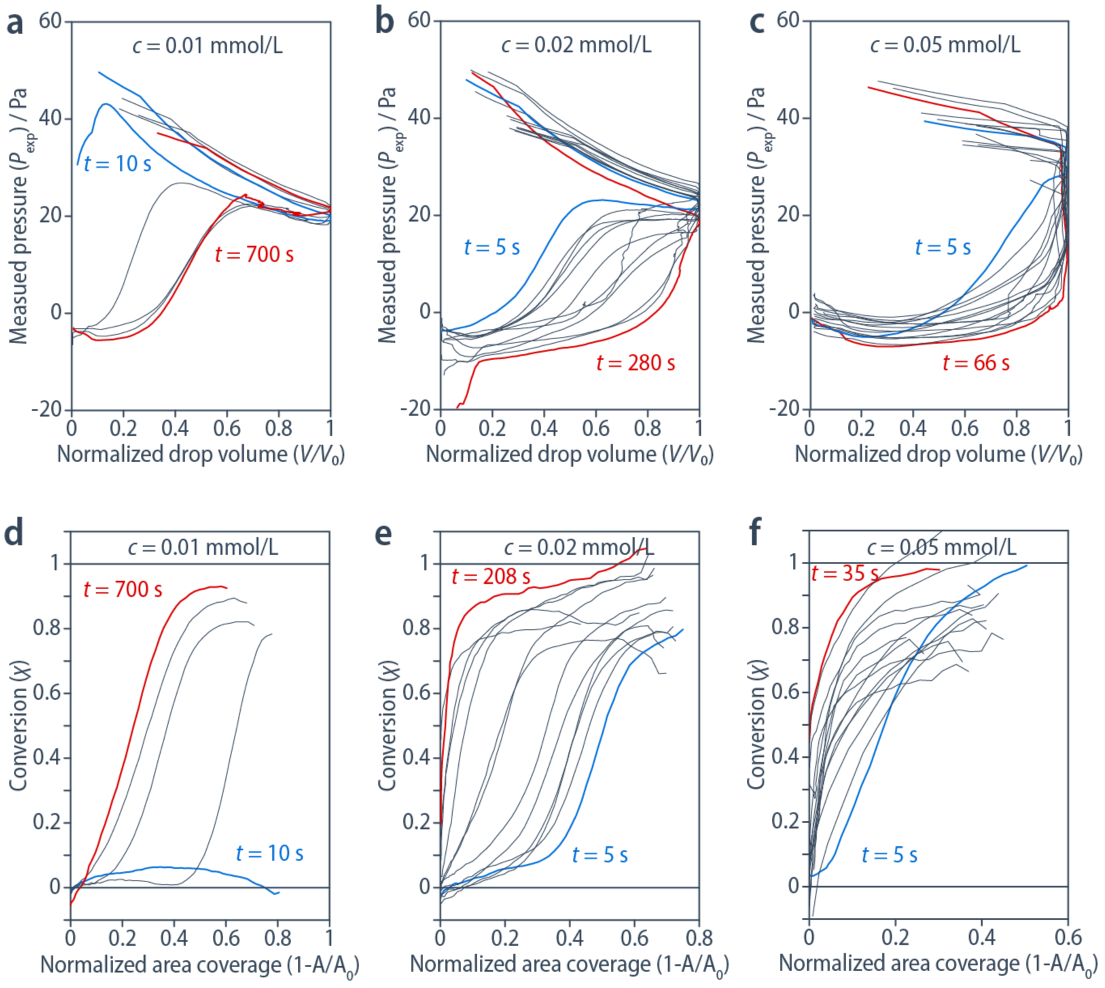

Supplementary Figure 6. Pressure-volume curves and conversion plots at all concentrations. **a–c**, Pressure-volume curves of the experimentally measured pressure inside the drop, P_{exp}, and the drop volume computed from the recorded drop shape V at different assembly times t and three different concentrations, c. At a given concentration, as the assembly time t is increased, the pressure inside the drop starts to reduce with an increasingly large volume during the deflation (*i.e.*, at an increasingly early stage of deflation). This effect is more pronounced at larger concentrations and at c = 0.05 mmol/L, the decrease starts immediately upon deflation. **d–f**, Overlay plots of conversion, $\chi = (P_{exp} - P_{\mathrm{is}})/(P_{\mathrm{is}} - P_{\mathrm{ip}})$, as a function of relative change of area coverage, $1-A/A_0$, at different assembly times, t, and three different concentrations, c. The pressure, P_{exp}, is measured experimentally with a pressure sensor, whereas P_{is} and P_{ip}, respectively, correspond to theoretical pressure predictions considering a constant number of surfactant molecules at the interface (liquid behavior) and a constant meridional perimeter (inextensible solid behavior). As t or c is increased, the conversion from liquid to solid behavior of the interface requires a smaller change in area coverage (less deflation).

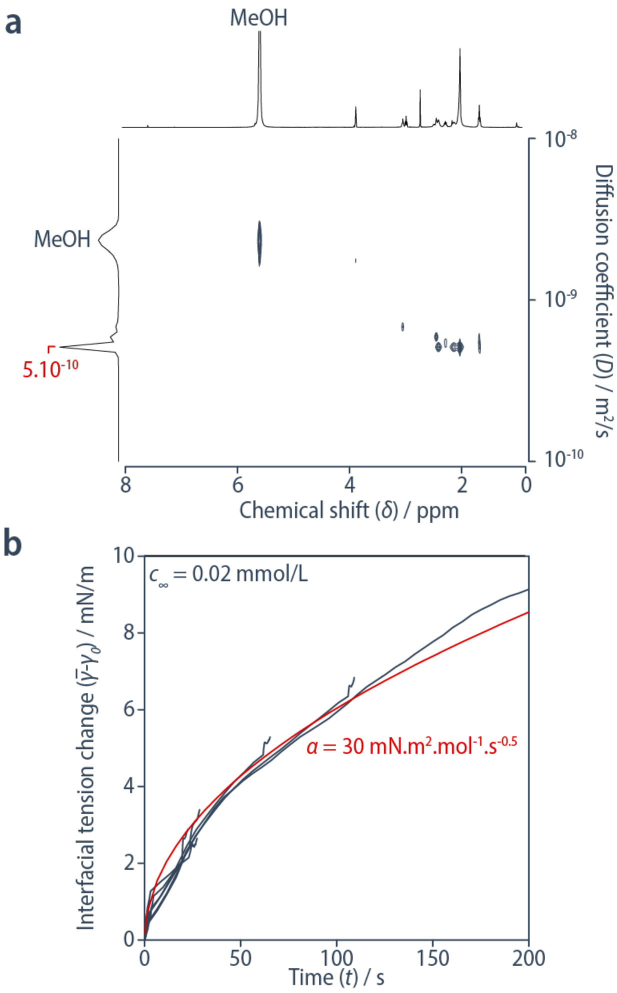

Supplementary Figure 7. Determination of the chemical potential K. **a**, 2D DOSY NMR spectroscopy of surfactant **1** recorded in $CDCl_3$ with 20% MeOH to increase the solubility of 1 (see Methods). **b**, Fitting of parameter α of the interfacial concentration change in the diffusion-limited regime.

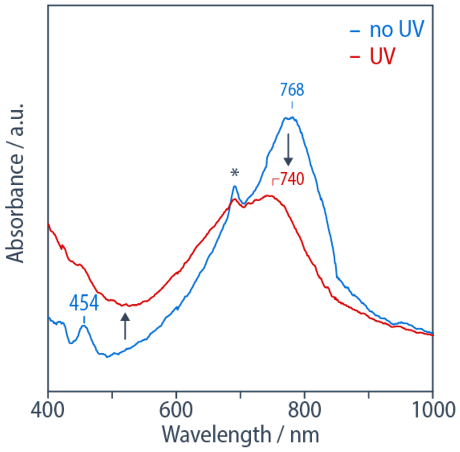

Supplementary Figure 8. UV-Vis absorption spectrum of the carbon nanoskin. The UV-vis spectrum is consistent with the initial formation of an extensively crosslinked poly(ene-yne) structure that progressively carbonizes (* equipment artefact)

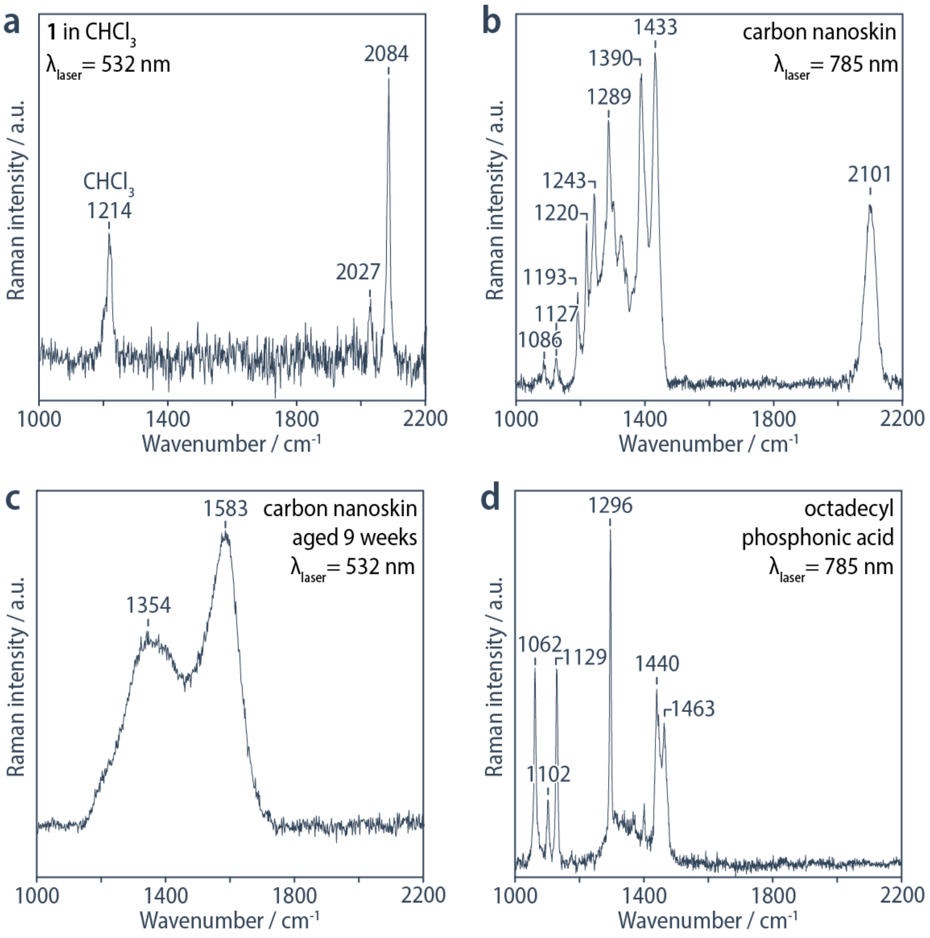

Supplementary Figure 9. Raman spectroscopy. **a**, The Raman spectrum of a solution of the carbon-rich surfactant **1** show the hexayne triple bond vibration at 2084 cm^{-1}. **b**, The Raman spectrum of an as-transferred carbon nanoskin exhibits a residual carbon-carbon triple bond at 2101 cm^{-1} as well as a series of overlapping peaks in the range of 1100–1450 cm^{-1} that can be attributed to the carbon-carbon double-bonds of an emerging D-band of amorphous carbon. **c**, The nanoskin after 9 weeks of ageing at the exhibits the characteristic D and G bands of amorphous carbon nanomaterials at 1354 and 1583 cm^{-1}, respectively, which are identical to the bands observed in UV-irradiated samples (Fig. 3a). **d**, A comparison to the Raman spectrum of a powder of octadecyl phosphonic acid powder shows that the bands observed at 1200–1450 cm^{-1} do not represent alkyl vibrations, with the possible exception of the peak at 1289 cm^{-1}, particularly because the Raman spectrum of the carbon nanoskin is taken at a laser excitation wavelength λ_{laser} = 785 nm, that is, in resonance with the optical absorption of the extended π-conjugated system.

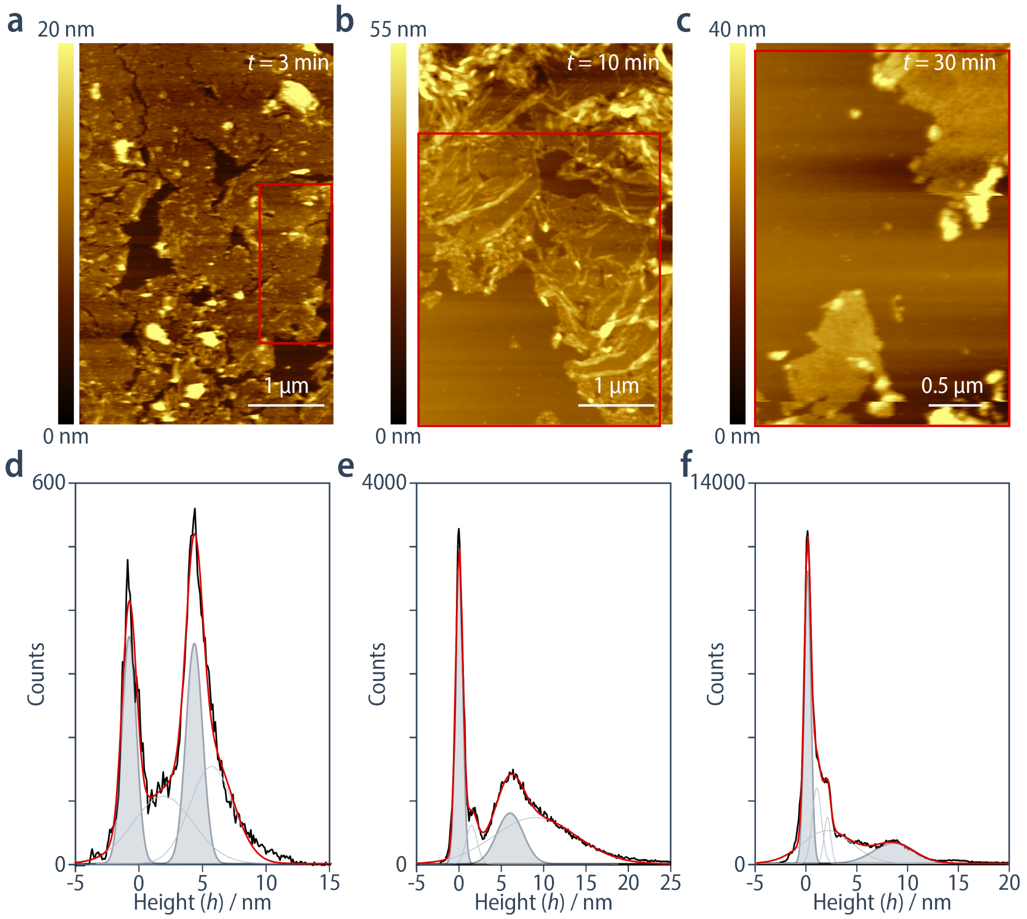

Supplementary Figure 10. Atomic force microscopy (AFM) imaging and histographic analysis as a function of assembly time, *t*. **a–c**, AFM micrographs on samples obtained by transfer of the carbon nanoskin to a polished silicon wafer after *t* = 3, 10, and 30 min, respectively. **d–f**, Deconvoluted histograms of the height signal extracted from cropped regions of the micrographs in a-c (red rectangles). From the fitting results of the component peaks corresponding to the substrate and film thickness (shaded in grey), the mean film thickness at each assembly time *t* was determined (see Supplementary Table 1).

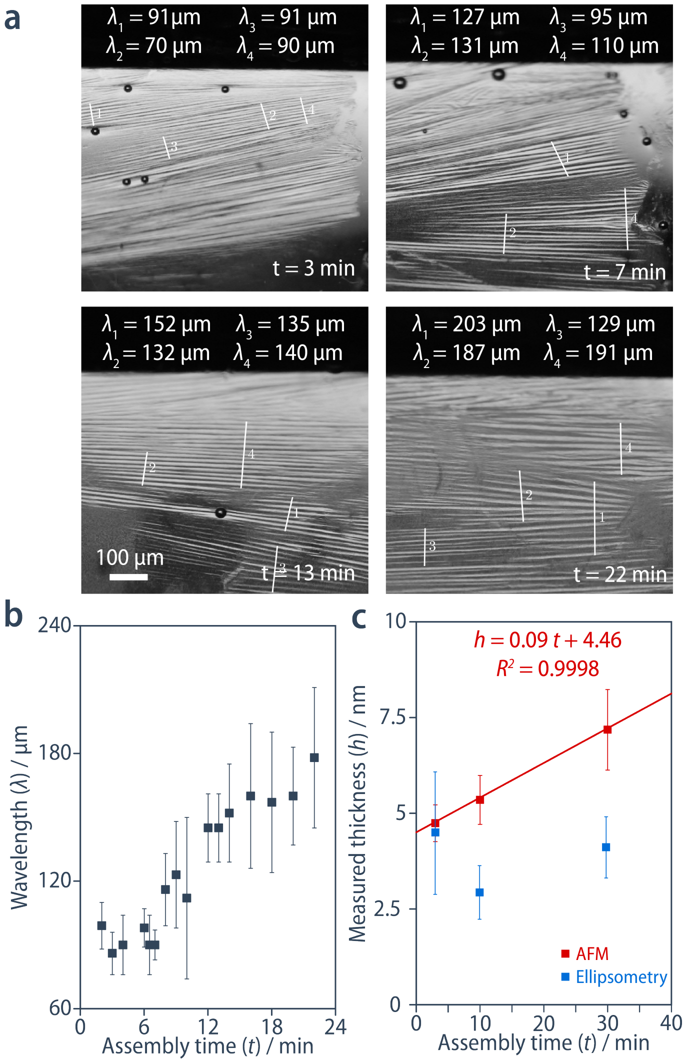

Supplementary Figure 11. Young's modulus characterization of the carbon nanoskin. Determination of the Young's modulus from the analysis of the elasto-gravitational wavelength of wrinkles upon compression of the nanoskin formed at a flat chloroform/water interface after different assembly times, *t*. **a**, Photographs of the wrinkles at the liquid-liquid interface. **b**, The elasto-gravitational wavelength λ of the wrinkles as a function of assembly time *t*, determined by image analysis. **c**, Evolution of film thickness with assembly time, *t*, according to histographic analysis of the AFM images (Supplementary Fig. 10) and ellipsometry.

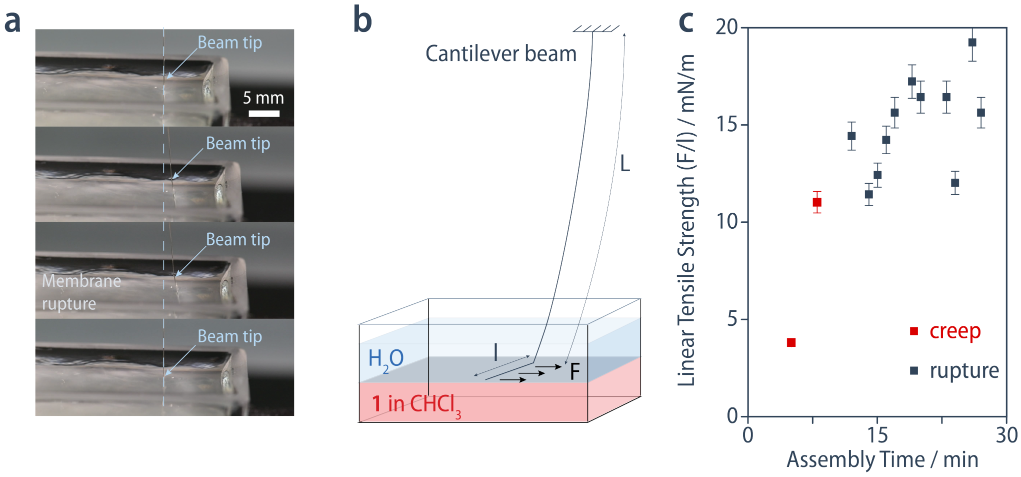

Supplementary Figure 12. Tensile strength determination. **a**, Photograph of the tensile strength measurement showing the increasing deflection of the cantilever with time as the bath is displaced towards the right. The blue dashed line marks the original vertical position of the cantilever without any applied force. **b**, Schematic of the experimental setup used to measure the tensile strength of the nanoskin (See Methods). **c**, The linear tensile strength increases with assembly time; the data points at short assembly times (red) represent an absence of a clear rupture of the nanoskin, but, instead, a viscoplastic creep through the interface.

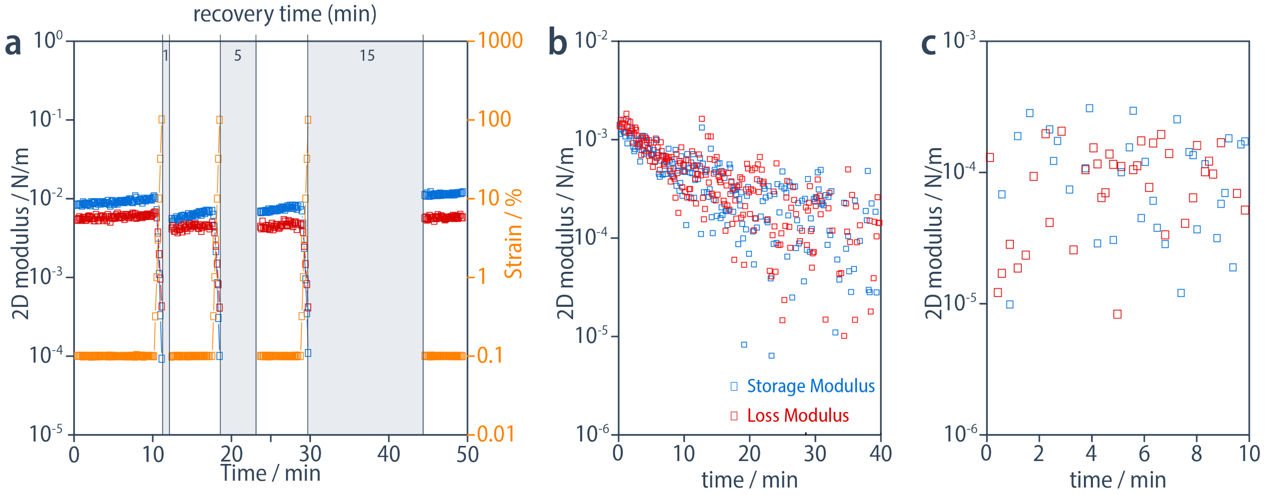

Supplementary Figure 13. Interfacial rheology. **a**, Self-healing behavior of the nanoskin at *c* = 0.02 mmol/L after 1 h assembly time at the oil-water interface (storage modulus, *G*', blue; loss modulus, *G*'', red; strain, orange). The nanoskin is ruptured upon a large increase in strain; the recovery increases with longer healing time (1 min, 5 min, 15 min), eventually reaching 120% the original storage modulus, *G*', and 90% of the original loss modulus, *G*'', indicating an even more elastic response after healing. **b**, Interfacial rheology measurement following the formation of the nanoskin at *c* = 0.02 mmol/L without any assembly time; the rupture irreversibly disrupts the mechanical integrity of the nanoskin. **c**, Interfacial rheology following the formation of the nanoskin at *c* = 0.01 mmol/L, after 1 h assembly time. The rheological properties of the skin cannot be assessed; similar observations are made in the case of bare $CHCl_3/H_2O$ interfaces and octadecyl phosphonic acid (*c* = 0.05 mmol/L) at $CHCl_3/H_2O$ interfaces.

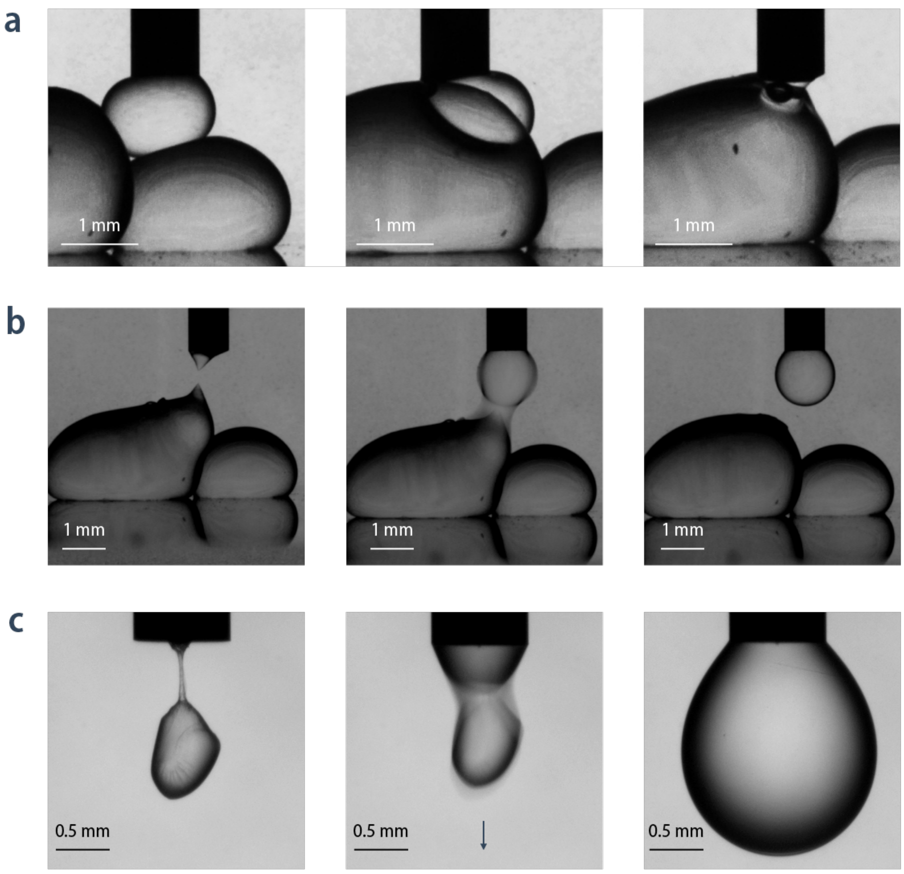

Supplementary Figure 14. Reconfigurability of the carbon nanoskin. **a**, Carbon-nanoskin-covered droplets of a chloroform solution of the carbon-rich surfactant **1** in a water bath only coalesce when they are pushed against each other, giving rise to complex shapes. **b**, Conical droplet which upon coalescence with a fresh droplet of chloroform solution recovers its liquid behavior and is reshaped into a spherical droplet. **c**, Inflation of a suspended droplet of chloroform solution in water. The freshly formed droplet shows the typical geometry of a fully liquid interface dominated by surface tension. Arrows indicate inflation. See Supplementary Movies S3, S5, and S6.

3. Supplementary Tables

Supplementary Table 1. Height determination from AFM image.

Assembly time, *t* (min)	Peak no	Center of gravity	FWMH	Thickness, *h* (nm)
3	1	-0.81	1.44	5.18 ± 1.53
	2	4.37	1.62	
10	1	-0.01	0.1	6.01 ± 1.89
	3	6.00	3.67	
30	1	0.06	0.79	8.67 ± 2.81
	5	8.61	4.82	

Supplementary Table 2. Young's modulus of carbon nanomaterials.

Material	Young Modulus (GPa)	Method	Reference
monolayer of graphene oxide flakes	100	wrinkling at the air-water interface	30
graphene oxide paper	40	macroscopic tensile testing	36
carbon nanomembrane	12–48	AFM	37
nanometer thick graphene oxide	208	AFM	38
