# Supplementary material for: Spontaneous formation of a self-healing carbon nanoskin at the liquid–liquid interface
Source: Nat Commun. 2022 Aug 23;13:4950. doi: 10.1038/s41467-022-31277-5 (PMC9399178; doi:10.1038/s41467-022-31277-5)
Supplement: Supplementary file 2 — Description of Additional Supplementary Files [file 41467_2022_31277_MOESM2_ESM.pdf]

Description of Additional Supplementary Files

File Name: Supplementary Movie 1
Description: Wrinkles at the interface. The nanoskin wrinkles upon interfacial area reduction. The reinjection of chloroform returns the droplet to a liquid state in which surface tension dominates (34 s). When the solidification occurs, the drop shape elongates (51 s). While the droplet is completely deflated into the needle and reinflated, the nanoskin remains intact.

File Name: Supplementary Movie 2
Description: Rapid inflation-deflation cycles. The nanoskin is spontaneously formed within seconds of the creation of the interface (4 s). It is mechanically robust enough to be cycled in and out of the syringe multiple times, sometimes leading to transient out-of-equilibrium elongated shapes (12 s).

File Name: Supplementary Movie 3
Description: Absence of spontaneous coalescence. The nanoskin prevents the coalescence of two droplets put in contact. Piercing the nanoskin with the needle (20 s) restores the capacity of droplets to coalesce.

File Name: Supplementary Movie 4
Description: Forced coalescence and channel formation. Two droplets are compressed to enforce their coalescence, leading to the formation of a channel. This channel can be used to transfer solution from the needle to the droplet (22 s). When the channel breaks, droplets with out-of-equilibrium conical shapes are formed (26 s).

File Name: Supplementary Movie 5
Description: Remodelling the interface. The forced coalescence forms a conical droplet (14 s). By injecting fresh chloroform solution (18 s), the liquid behavior of the conical droplet is restored, resulting in a round-shaped droplet.

File Name: Supplementary Movie 6
Description: Suspended droplet. A droplet covered by the carbon nanoskin is suspended from the needle by a small nanoskin filament. This filament can be used to inject fresh solution and recover a droplet with a fully liquid interface.

File Name: Supplementary Movie 7
Description: Collapsed droplet. The chloroform solution of a deposited droplet is removed to demonstrate the presence of the nanoskin. A gas bubble becomes trapped in the process, indicating the impermeability of the nanoskin.
